# RNA-seq profiling identified a three-lncRNA panel in serum as potential biomarker for muscle-invasive bladder cancer

**DOI:** 10.3389/fonc.2024.1451009

**Published:** 2024-12-16

**Authors:** Xiumei Jiang, Ailin Qu, Shoucai Zhang, Shuchao Jin, Lishui Wang, Yi Zhang

**Affiliations:** ^1^ Department of Clinical Laboratory, Qilu Hospital, Shandong University, Jinan, Shandong, China; ^2^ Department of Urology, Qilu Hospital, Shandong University, Jinan, Shandong, China

**Keywords:** muscle-invasive bladder cancer, serum, biomarker, diagnosis, lncRNA

## Abstract

**Background:**

Preoperative determination of muscular infiltration is crucial for appropriate treatment planning in patients with muscle-invasive bladder cancer (MIBC). We aimed to explore early diagnostic biomarkers in serum for MIBC in this study.

**Methods:**

The expression profiles of long noncoding RNA (lncRNA) were initially screened by high-throughput sequencing and evaluation of potential lncRNAs were conducted by two phases of RT-qPCR assays using serum samples from 190 patients with MIBC and 190 non-muscle-invasive BC (NMIBC) patients. Multivariate logistic regression analysis was applied to establish a diagnostic signature with high accuracy and Fagan’s nomogram was plotted to promote clinical application. Bioinformatics analysis was used to determine the potential miRNA-mRNA binding of candidate lncRNAs.

**Results:**

We identified three differentially expressed lncRNAs (LINC00565, LINC00592 and NDUFA6-AS1) and established a 3-lncRNA panel which demonstrated high diagnostic accuracy for MIBC with an AUC of 0.903 (95% CI: 0.850-0.942) and 0.875 (95% CI: 0.802-0.928) in the training and validation set. Moreover, construction and assessment of Fagan’nomogram demonstrated that the 3-lncRNA panel could exhibit practical and helpful values for clinical use. Finally, a network map based on LINC00565 was constructed and we found that the expression of miR-143-5p and miR-4516 were significantly correlated with LINC00565 in MIBC.

**Conclusion:**

Our findings indicated that the constructed 3-lncRNA panel in serum showed favorable diagnostic capacity and might serve as promising non-invasive biomarkers in the early diagnosis of MIBC.

## Introduction

Bladder cancer (BC) is one of the most common malignancies of the urinary system, accounting for 573,000 new cases and 213,000 deaths worldwide by 2020 (1). At initial diagnosis, approximately one-third of patients already present with muscle-invasive bladder cancer (MIBC) and exhibit unfavorable outcomes, with a 5-year overall survival (OS) rate of less than 10% (2, 3). With respect to the invasion depth of lesions, patients with MIBC usually require systemic therapies, including radical cystectomy, pelvic lymph node dissection and neoadjuvant chemotherapy (4). Thus, precise diagnosis of MIBC is of particularly importance for the choice of clinical treatment (5). However, the accuracy of cystoscopy and histological evaluation is unsatisfactory, presenting inconsistency with the postoperative stage based on the whole bladder at times (6). It is estimated that nearly 50% of patients undergoing radical cystectomy (RC) are clinically understaged (7). Current deficiencies of the invasive approach for clinical staging lie in undersampling of muscularis propria (MP) on transurethral resection (TUR) or delays from diagnosis of MIBC to RC (8). Moreover, the value of traditional imaging and urine cytology in assessing local invasion is limited owing to its low accuracy (9, 10). Therefore, reliable non-invasive biomarkers are urgently needed to assess the muscle-invasive status of BC.

Long noncoding RNAs (lncRNAs) are a class of RNA transcripts longer than 200 nucleotides that have recently emerged as a novel hot area of research in cancer (11). Dysregulated lncRNAs can function as molecular sponges in interactions with microRNAs (miRNAs) and mRNAs and exert crucial effects on carcinogenesis through complex signal-regulating networks (12, 13). Recent studies have revealed that lncRNAs can exist stably in serum and play potential roles in diagnosis of cancer (14–16). In case of MIBC, Zhou et al. (17) found that LUCAT1 could implicate pathogenesis by targeting miR-199a-5p and miR-199b-5p and was upregulated in serum. Although we previously identified distinct lncRNA panels in serum for diagnosis of certain types of cancer, including BC (18–20), the unique expression profiles of lncRNAs as biomarkers for the diagnosis of MIBC have not been comprehensively identified.

In the present study, we systematically screened serum lncRNA profiles by sequencing and two independent phases of RT-qPCR assays. Three differentially expressed lncRNAs were identified and incorporated into a specific signature with high diagnostic accuracy for MIBC. Moreover, a network map based on lncRNAs and miRNAs was further estimated and assessed. Our findings may lay foundation for serum lncRNAs as potential biomarkers for diagnosing muscle-invasive status and provide a step towards precision surveillance for MIBC.

## Methodology

### Study design

The study was divided into three phases (**Supplementary Figure S1**). To ensure sample balance between MIBC and NMIBC, a total of 190 MIBC patients and 190 NMIBC patients were enrolled and they were randomly allocated into three phases following a 4:9:6 ratio. In the screening phase, serum samples from 40 patients with MIBC and 40 non-muscle-invasive BC (NMIBC) patients were respectively divided into four subgroups and independently subjected to sequencing to select potential lncRNAs. In the training phase, candidate lncRNAs were firstly tested and selected by RT-qPCR in 30 MIBC patients and 30 NMIBC patients. The different expressions of 3 lncRNAs between NMIBC and MIBC were further confirmed in additional groups of 60 MIBC patients and 60 NMIBC patients. Meanwhile, 90 healthy controls were selected to evaluate the expression of lncRNAs in serum. Afterwards, a combination of 90 MIBC and 90 NMIBC patients was used to construct a diagnostic panel. Using the regression coefficients of the multivariate model to weigh the power of each lncRNA, a formula for the lncRNA panel was established to diagnose MIBC. In the validation phase, the accuracy of the constructed panel was validated using serum samples from another cohort of 60 MIBC patients and 60 NMIBC patients. Additionally, the expression levels of lncRNAs in tumor tissues from 15 MIBC patients in the validation cohort were explored, and a network map based on lncRNAs was constructed. Patients with BC and healthy controls were recruited from the Department of Urology and Healthy Physical Examination Center of Qilu Hospital, Shandong University. This study was approved by the Clinical Research Ethics Committee of the Qilu Hospital, Shandong University (KYLL-202107-102), and written informed consent was obtained from each participant.

### Specimen collection and preparation

Approximately 5 mL venous blood samples from participants were collected before radical cystectomy and/or TUR and separated by centrifugation at 4,000rpm for 10min within 2h, followed by a second centrifugation at 12,000rpm for 15min. Supernatant serum was stored at -80°C. Fresh tumor tissues and adjacent noncancerous tissues (≥3cm away from the tumor) were immediately frozen in liquid nitrogen and stored at -80°C.

### Illumina high-throughput sequencing

Transcriptome HTS and subsequent bioinformatic analyses were conducted by CloudSeq Biotech (Shanghai, China). Briefly, RNA was isolated using Onestep-Lysis™ Serum/Plasma RNA Kit (NOBELAB, China) and rRNAs were removed using NEBNext rRNA Depletion Kit (New England Biolabs, Inc., USA). RNA purity was evaluated by Qubit Fluorometer. The NEBNext^®^ Ultra™ II Directional RNA Library Prep Kit (New England Biolabs, Inc., USA) and BioAnalyzer 2100 system (Agilent Technologies, USA) were then used to construct and quantify/qualify RNA libraries, respectively. Subsequently, 10-pM RNA libraries were denatured as single‐stranded DNA molecules, captured on Illumina flow cells, amplified *in situ* as clusters, and finally sequenced for 150 cycles on Illumina NovaSeq 6000 Sequencer.

### LncRNA sequencing analysis

Following image and base recognition, original reads were harvested by sequencing and quality control was performed based on Q30. Cutadapt software was used to 3′ adaptor-trimming and low-quality removal. High-quality clean reads were selected and aligned to the human reference genome (UCSC HG19) using hisat2 software. Cuffdiff software was employed to obtain gene level fragments per kilobase of exon per million (FPKM) reads as lncRNA expression profiles. Fold change (FC) and *p* values of the comparison between the 4 NMIBC samples and 4 MIBC samples were calculated based on the FPKM data. LncRNAs showing fold changes ≥2.0 and *p*<0.05 were selected as differentially expressed. GO and KEGG analyses were applied to predict functions of selected lncRNAs.

### Analysis of lncRNA-miRNA-mRNA network

TargetScan and Miranda software were used to predict the miRNA-binding sites and target mRNAs. The top five predicted miRNAs and five most likely downstream genes for each miRNA were selected. Cytoscape was used to establish a lncRNA-miRNA-mRNA network to visualize the interactions between these molecules.

### Quantification of lncRNA by RT-qPCR

Total RNA was isolated from the serum and tissues/cells using TRIzol LS and TRIzol reagent (Invitrogen, Carlsbad, CA, USA). The quantity of RNA were measured using NanoDrop spectrophotometer. To further validate the quality of RNA isolation, we also used Onestep-Lysis™ Serum/Plasma RNA Kit for RNA isolation and Qubit fluorometer for concentration measurement in 30 MIBC patients and 30 NMIBC patients in the training cohort. RT reactions were conducted using a PrimeScript^®^ RT reagent kit (Takara, Dalian, China) in a total volume of 20μL (1 μg of template RNA, 4 μL of 5× PrimeScript Buffer, 1 μL of PrimeScript RT Enzyme Mix I, 1 μL of Oligo dT Primer, and RNase-free dH_2_O). The reaction was incubated at 37°C for 30min, followed by 85°C for 5s and 4°C for 60min. Quantitative PCR reaction for lncRNA was carried out using reagents contained 2μL of cDNA, 12.5μL of SYBR Premix Ex Taq, 0.5μL of ROX Reference Dye α, 1μL of forward primer, 1 μL of reverse primer and 8μL of RNase-free dH_2_O. Reactions were then incubated at 95°C for 30 s, followed by 42 cycles of 95°C for 5s and 60°C for 34s. miRNA quantification was performed as previously described (21). RT-qPCR assays were performed using a CFX96™ Real-Time PCR Detection System (Bio-Rad Laboratories, Hercules, CA, USA). GAPDH and U6 were used as endogenous controls for lncRNAs and miRNAs, respectively. The relative gene expression levels were calculated using the 2^-ΔΔCt^ method. lncRNAs with Ct values>35 and a detection rate<75% were excluded. Primers used for RT-qPCR are listed in **Supplementary Table S1**.

### Cell culture

BC cell lines T24 and 5637 and the human immortalized uroepithelium cell line SV-HUC-1 were purchased from the Cell Bank of the Chinese Academy of Sciences (Shanghai, China). T24 and 5637 cells were cultured in RPMI 1640 medium (Gibco, USA), SV-HUC-1 cells were cultured in F-12K medium (Gibco, USA) supplemented with 10% fetal bovine serum (BI, Israel) in a humidified atmosphere of 5% CO_2_ at 37°C.

### Statistical analysis

The “sample” function in R language is utilized for random sampling. The data distribution of each group was measured using the Kolmogorov-Smirnov test. The nonparametric Mann–Whitney U test was performed to compare the differential expression of lncRNAs between the different groups. Logistic regression analysis was performed using MATLAB software (MATLAB R2014a). The area under the ROC curve (AUC) was established using MedCalc 9.3.9.0 to discriminate subjects with or without muscle-invasive tumors. Fagan’s nomogram was used to help clinicians calculate the probability of MIBC according to the diagnostic test results. The correlation between two variables was measured using Spearman’s correlation analysis. SPSS software (version 18.0, Chicago, IL, USA) and R software (version 3.2.3; http://www.Rproject.org) were used to analyze all other data. *P*<0.05 was considered as statistically significant.

## Results

### Characteristics of participants

All patients with MIBC/NMIBC were initially diagnosed according to the 2002 UICC for International Cancer Control TNM classification and showed no evidence for other types of tumor. The most relevant clinical features of BC including sex, age, histological grade, stage, lymph node metastasis, vascular invasion and tumor size were collected from medical records of each patient. Categorical variables of these characteristics were set in accordance with literature (22, 23), and all BC patients were matched with these features. Variables were presented as numbers (%) and analyzed using Pearson’s chi-squared test or Fisher’s exact test as appropriate. There were no differences in clinical characteristics between the NMIBC cohorts and the MIBC cohorts in the three phases (all at *p*>0.05). Moreover, the distribution of patients with different features were all similar between the training set and the validation set (all at *p*>0.05). The clinical characteristics of 190 MIBC patients and 190 NMIBC patients were summarized in **Supplementary Table S2**.

### Expression profiling of lncRNAs in MIBC by HTS

Transcriptomic analyses using Illumina NovaSeq 6000 sequencing were performed to assess the differences in lncRNA expression between 4 MIBC samples and 4 NIMBC samples. RNA-seq of eight cDNA libraries yielded over 50 million raw reads, with most being clean reads, and over 88.63% of clean reads mapped perfectly to the reference human genome (**Supplementary Table S3**). Total of 5712 lncRNAs were detected (**Supplementary Figure S2A, B**). According to the selection criteria, 205 lncRNAs were found to be significantly upregulated and 300 lncRNAs were downregulated in MIBC (**Supplementary Figure S2C**).

**Figure 1:**
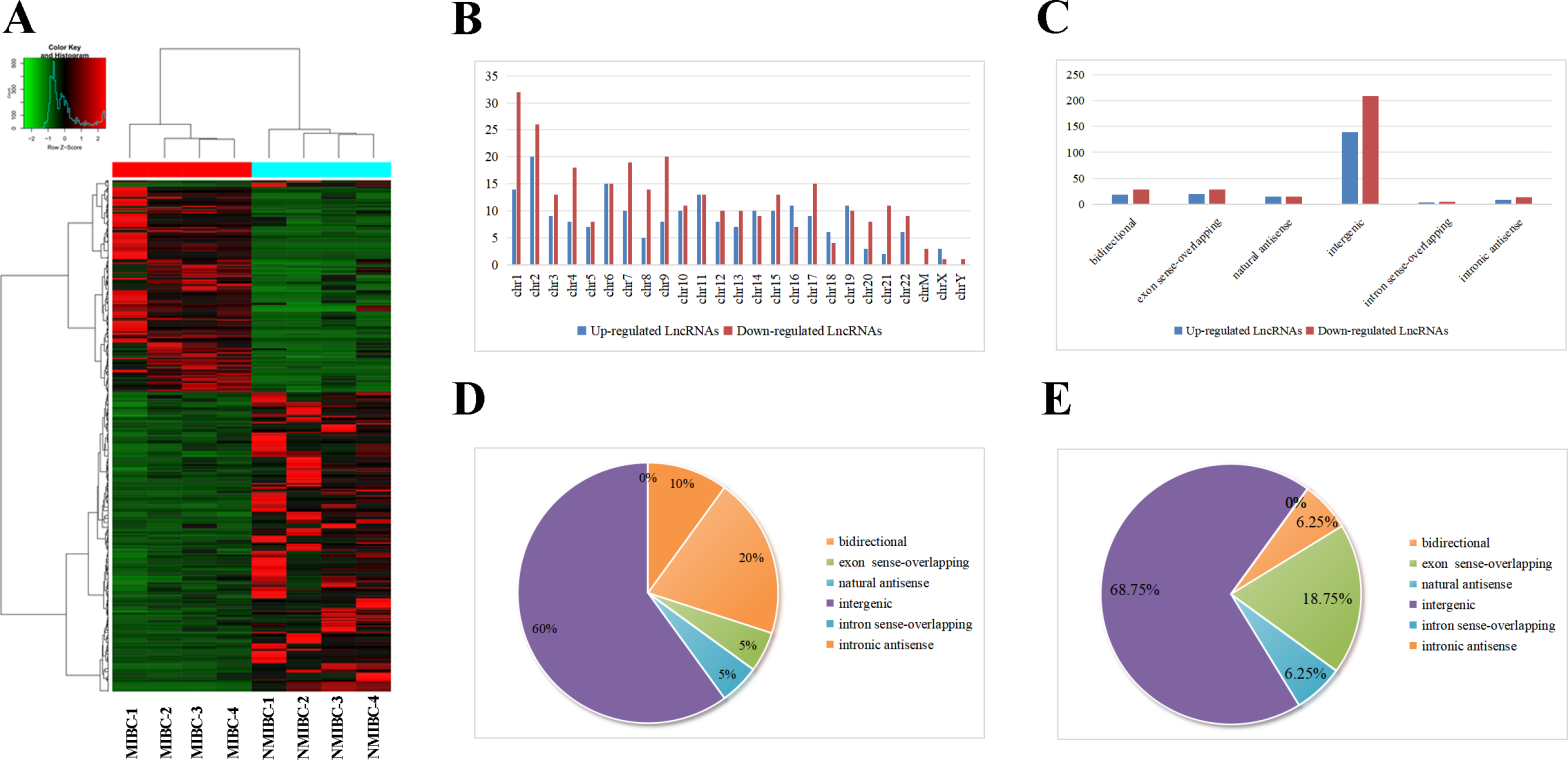
Analysis of upregulated and downregulated lncRNAs in MIBC by HTS. **(A)** Hierarchical clustering depict differences in lncRNA expression between NMIBC group and MIBC group. **(B)** Distributions of dysregulated lncRNAs in chromosomes, in which chromosome 2 and chromosome 1 contained the maximum quantity of upregulated and downregulated lncRNAs respectively. **(C)** Category classifications of the upregulated and downregulated lncRNAs, in which intergenic origin accounts for the majority. **(D)** Classification of the upregulated lncRNAs in chromosome 2 based on their genomic origin. **(E)** Classification of the downregulated lncRNAs in chromosome 1 based on their genomic origin.

Hierarchical clustering demonstrated significant differentially expressed lncRNAs (**Figure 1A**). All these lncRNAs were widely distributed among all chromosomes, with chromosome 2 and chromosome 1 containing the largest number of upregulated lncRNAs and downregulated lncRNAs, respectively (**Figure 1B**). Moreover, intergenic origin was the most common category of forming information of both upregulated and downregulated lncRNAs (**Figure 1C**). In chromosome 2 and chromosome 1, intergenic origins both account for the majority category based on how they were produced (**Figures 1D, E**). Based on these findings, the upregulated lncRNAs in chromosome 2 overlapped with the upregulated intergenic lncRNAs whereas the downregulated lncRNAs in chromosome 1 overlapped with the downregulated intergenic lncRNAs. Finally, the top 10 most upregulated intergenic lncRNAs in chromosome 2 and top 10 most downregulated intergenic lncRNAs in chromosome 1 were shown in **Supplementary Table S4**, which may lay the basis for discerning the biological differences of lncRNAs in MIBC. The sequencing data were uploaded to the NCBI Gene Expression Omnibus database (accession no. GSE255069).

### Predicted functions of differentially expressed lncRNAs in MIBC

**Figure 2:**
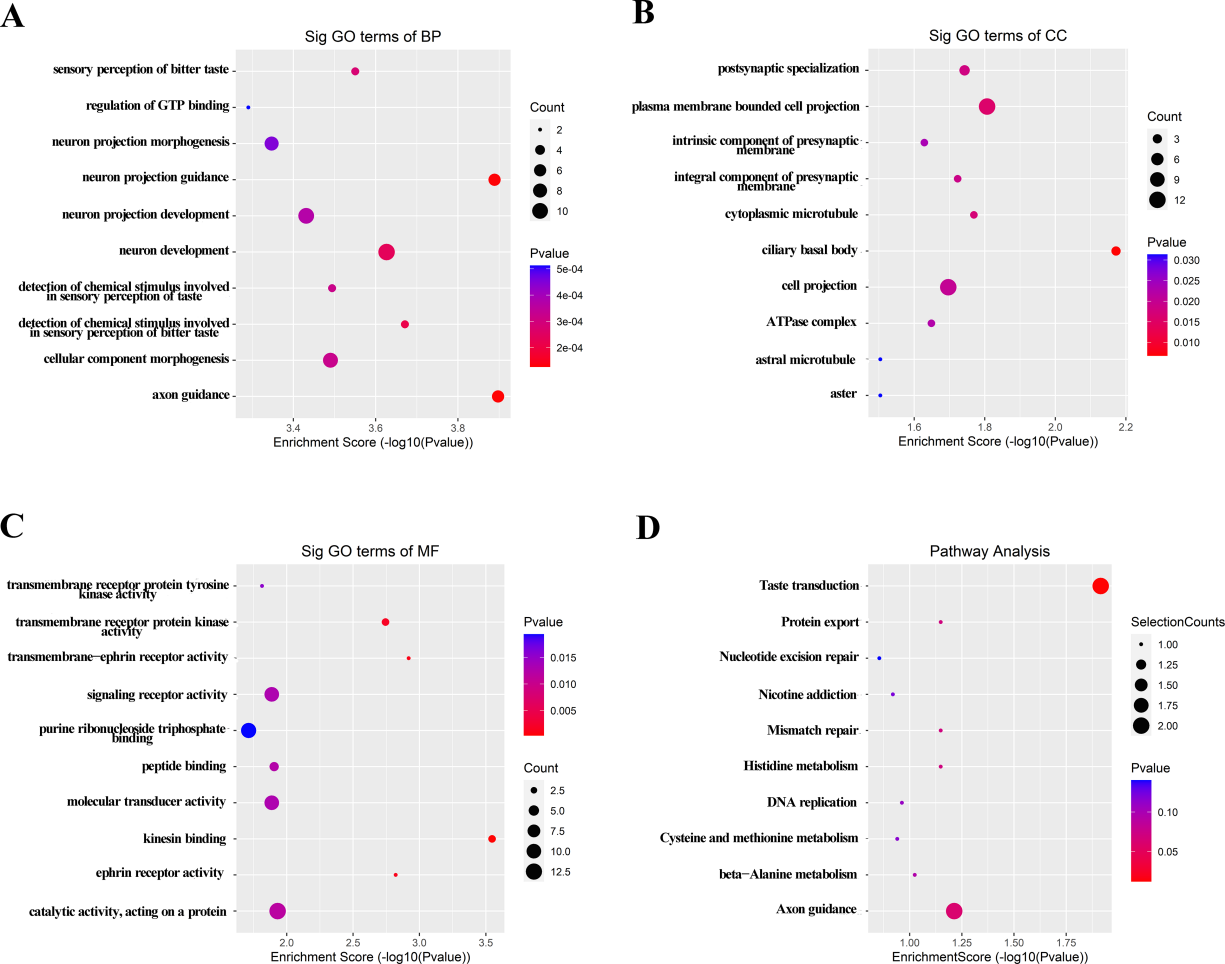
GO and KEGG signaling pathway analysis of the parent gene regulated by over-expressed lncRNAs in serum of MIBC. GO analysis included BP **(A)**, CC **(B)** and MF **(C)**. **(D)** Possible enriched pathway terms related to the upregulated lncRNAs. *P* < 0.05 was termed as significant.

Considering the contributions of parental genes to biological processes (BP), cellular components (CC), molecular functions (MF), and pathways, we conducted GO and KEGG analyses of 205 upregulated lncRNAs to predict their potential functions. GO analysis revealed that axon guidance, neuron projection guidance, and detection of chemical stimulus involved in sensory perception of bitter taste were the top three in terms of BP (**Figure 2A**). For CC, the three most significant terms refer to the ciliary basal body, plasma membrane bounded cell projection, and cytoplasmic microtubule (**Figure 2B**). With regard to MF, three significantly enriched terms were kinesin binding, transmembrance-ephrin receptor activity, and ephrin receptor activity (**Figure 2C**). KEGG analysis revealed that the taste transduction may be the most significantly affected signaling pathways (**Figure 2D**). GO and KEGG analyses of the downregulated lncRNAs also indicated potential functions or pathways, as shown in **Supplementary Figure S3**.

### Identification of differentially expressed lncRNAs in serum of MIBC by RT-qPCR assays

**Figure 3:**
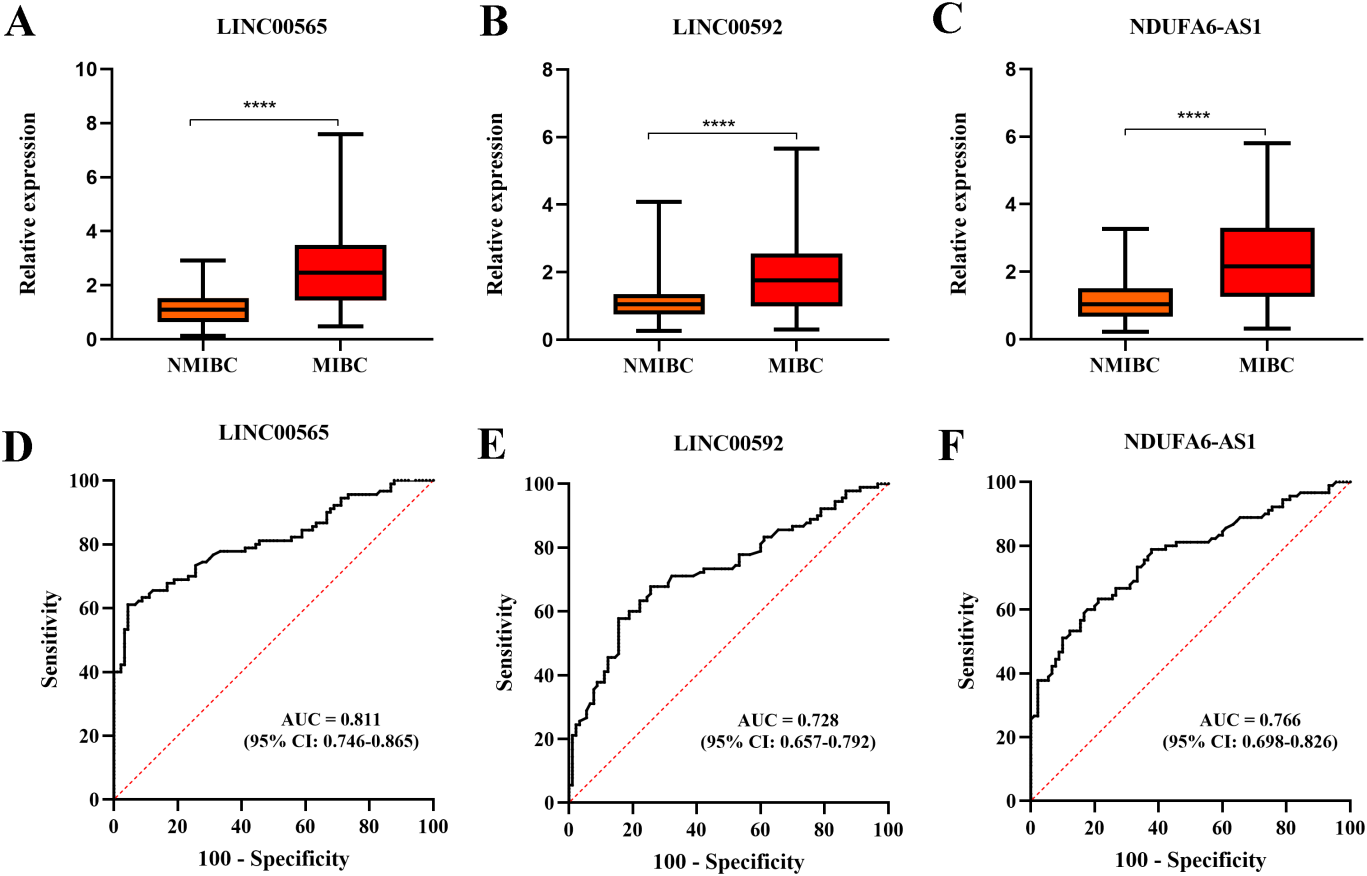
The Box-whisker plots and ROC plots represent for LINC00565, LINC00592, and NDUFA6-AS1 in the training set. **(A–C)** Differential expression patterns of LINC00565 **(A)**, LINC00592**(B)**, and NDUFA6-AS1 **(C)** between MIBC and NMIBC using RT-qPCR assay. **(D–F)** ROC curve analysis for diagnosis of MIBC using LINC00565 **(D)**, LINC00592 **(E)**, and NDUFA6-AS1**(F)**, ****p<0.0001.

Considering that downregulated lncRNAs may not be easy to detect, we selected the top 20 upregulated lncRNAs in HTS for RT-qPCR analysis on initial 30 MIBC patients and 30 NMIBC patients. Our results showed that four lncRNAs were not detectable in NMIBC/MIBC, and 13 lncRNAs demonstrated similar expressions between NMIBC and MIBC (all at *p*>0.05). Only three lncRNAs, including LINC00565 (NR_047495), LINC00592 (ENST00000549830), and NDUFA6-AS1 (ENST00000416037), showed differential expression patterns in MIBC (all at *p*<0.05), which were selected for further validation (**Supplementary Figure S4**). Moreover, we are surprising to find that these three lncRNAs were also differently expressed in MIBC compared to healthy controls. We then expanded the sample size to 90 MIBC patients and 90 NIMBC patients and confirmed the differential expression of three lncRNAs in MIBC in the training set (**Table 1**). We found that LINC00565, LINC00592, and NDUFA6-AS1 were significantly upregulated in MIBC (**Figures 3A–C**; **Supplementary Figure S5**). The corresponding AUCs of the three lncRNAs for MIBC were 0.811 (95% confidence interval (CI):0.746-0.865, with sensitivity (SN) of 61.1% and specificity (SP) of 95.6%), 0.728 (95% CI:0.657-0.792, with SN of 67.8% and SP of 74.4%) and 0.766 (95% CI: 0.698-0.826, with SN of 58.9% and SP of 83.3%), respectively (**Figures 3D–F**; **Supplementary Table S5**).

**Table 1:** Relative expression of candidate lncRNAs in MIBC and NMIBC patients in the training set and validation set.

LncRNA	Training set			Validation set		
	NMIBC (n=90)	MIBC (n=90)	*p*-Value	NMIBC (n=60)	MIBC (n=60)	*p*-Value
LINC00565	1.11 (0.65-1.53)	2.47 (1.44-3.49)	<0.0001	1.09 (0.64-1.59)	2.28 (1.42-3.50)	<0.0001
LINC00592	1.05 (0.76-1.35)	1.77 (0.99-2.56)	<0.0001	1.12 (0.65-1.47)	1.57 (0.91-2.73)	<0.001
NDUFA6-AS1	1.05 (0.67-1.51)	2.16 (1.26-3.30)	<0.0001	1.10 (0.71-1.44)	1.69 (0.99-2.48)	<0.0001

Data were presented as median (interquartile range).

**Figure 4:**
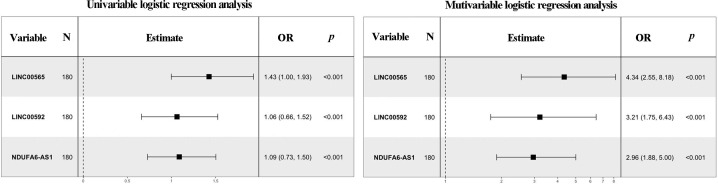
Forest plot summary of analyses of muscle-invasive status. Univariate and multivariate logistic regression analysis of the three lncRNAs for MIBC diagnosis in the training set. The squares on the transverse lines refer to the odds ratio (OR), and the transverse lines indicate the 95% CI.

As expected, the alteration patterns of these lncRNAs in the validation set were consistent with those in the training set, and the corresponding AUCs of the three lncRNAs were 0.787 (95% CI:0.703-0.856, with SN of 61.7% and SP of 88.3%), 0.664 (95% CI:0.572-0.748, with SN of 53.3% and SP of 80.0%) and 0.726 (95% CI: 0.637-0.803, with SN of 55.0% and SP of 90.0%), respectively (**Table 1**; **Supplementary Figure S6**). Logistic regression analysis revealed that these lncRNAs could serve as independent diagnostic factors for MIBC (**Figure 4**).

As there were fewer females than males in this study, we also analyzed the expression of three lncRNAs between females and males. No significant influences on expression of the three lncRNAs for the sex were found (**Supplementary Figures S7–S9**). Moreover, we found that the expression patterns and AUCs of three lncRNAs by RT-qPCR assays based on Qubit and NanoDrop were similar, which confirmed the quality and quantity of RNA (**Supplementary Figure S10**). All these results could provide new basis for the reliability of the RT-qPCR data in this study.

### Establishment of the 3-lncRNA panel for MIBC

**Figure 5:**
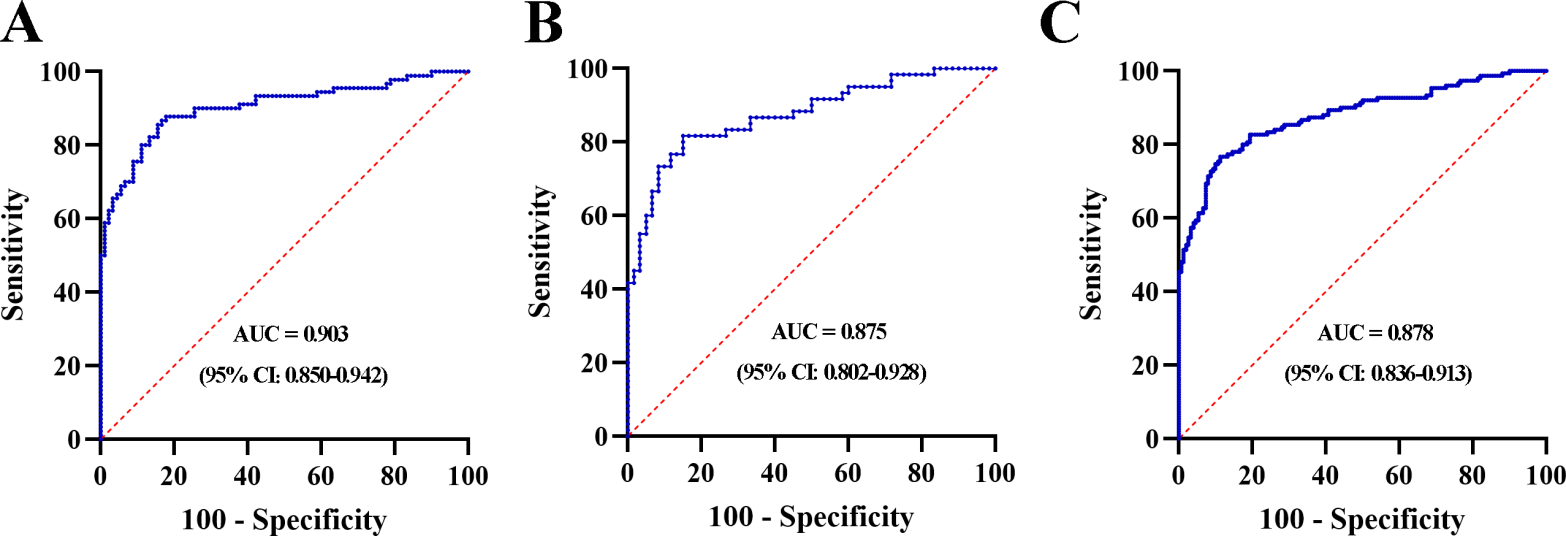
ROC curve analysis for the 3-lnRNA panel in the training set **(A)**, validation set **(B)** and combination set **(C)**.

A stepwise logistic regression model was established to determine the probability of diagnosing MIBC in the training set. The lncRNA-based panel was constructed using the following formula: Logit (P = MIBC) =-5.781+ (1.467 × LINC00565) + (1.166 × LINC00592) + (1.085 × NDUFA6-AS1). ROC curve analysis revealed that the AUC of the three-lncRNA panel was 0.903 (95% CI: 0.850-0.942), with SN of 86.7% and SP of 83.3% (**Figure 5A**). The diagnostic efficiency of the 3-lncRNA panel appears superior to the lncRNA alone.

### Validation of the 3-lncRNA panel for MIBC

The lncRNA-based panel obtained from the training set was used to calculate the probability of being diagnosed as MIBC in the validation set. Similar to the training set, the AUC of the lncRNA panel was 0.875 (95% CI: 0.802-0.928, **Figure 5B**). We further pooled MIBC patients from the training and validation sets, and the combined results showed that the constructed lncRNA panel could diagnose MIBC with an AUC of 0.878 (95% CI: 0.836-0.913) (**Figure 5C**). Furthermore, the diagnostic accuracy of the lncRNA panel for different stages of T2, T3, and T4 were analyzed and relative data were shown in **Supplementary Figure S11**.

### Construction and assessment of Fagan’nomogram to calculate the probability of MIBC based on 3-lncRNA panel

**Figure 6:**
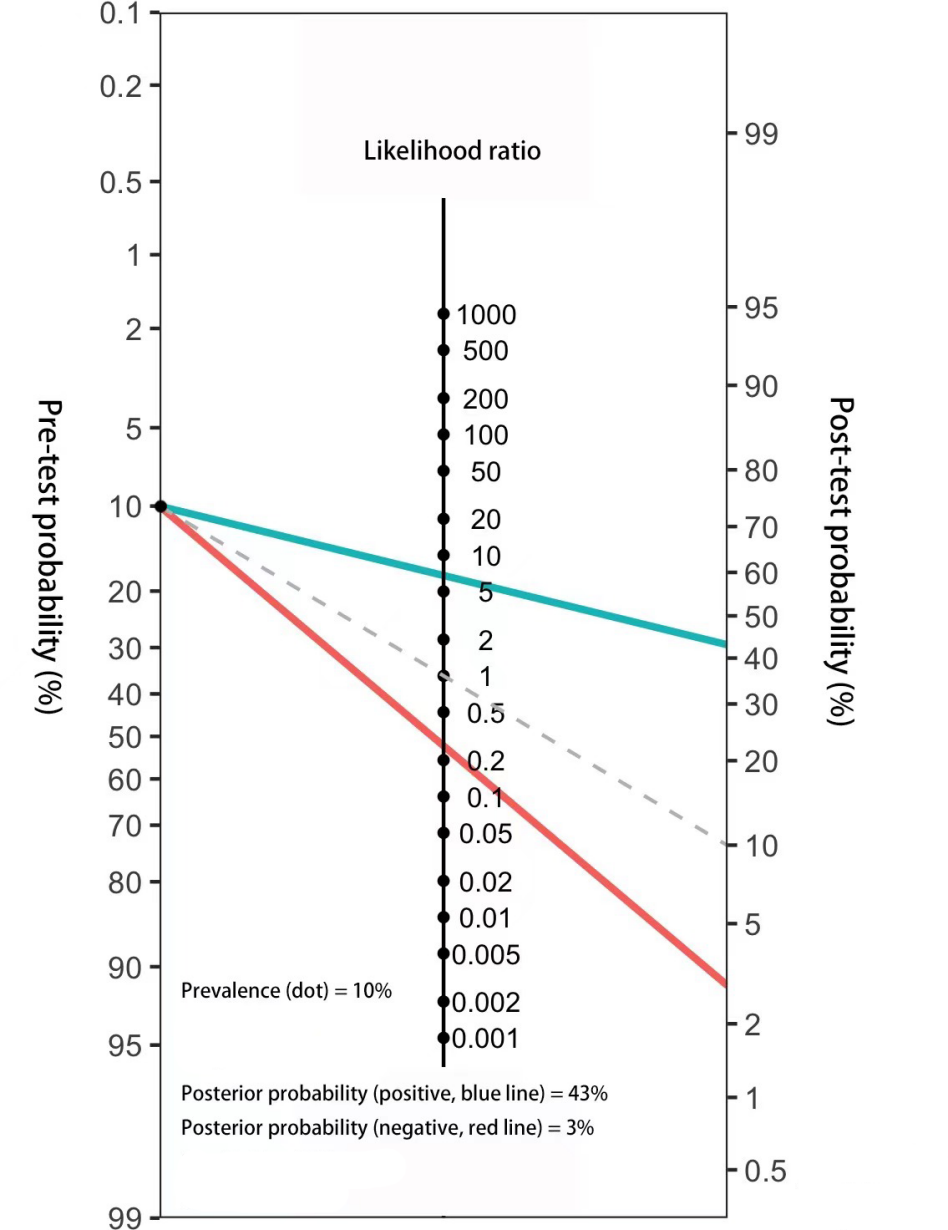
Fagan’s nomogram for the assessment of the probability that an individual has MIBC according to the 3-lncRNA panel.

The results of the combination set showed the 3-lncRNA Panel could diagnose MIBC with SN of 0.767 (95% CI: 0.691–0.832) and SP of 0.887 (95% CI: 0.825–0.933). To make this finding clinically available, Fagan’s nomogram was constructed to assist clinicians in using panel values to evaluate the probability of a patient having MIBC. The pre-test probability of the MIBC was set at a hypothetical value of 10%. The positive likelihood ratio (+LR) of the panel was 6.788 and the negative likelihood ratio (-LR) was 0.263. The pre-test probability and LR values are marked on the left and middle axes, respectively, followed by a drawn line from the two marked points along the right axis. Thus, the post-test probability was the point at which the line intersects with the right axis. As shown in **Figure 6**, if a patient had a positive panel result, the post-test probability that he truly had MIBC would increase to 43% (blue line). However, if one had a negative panel result, the post-test probability would decrease to 3% (red line). These data demonstrate that our 3-lncRNA panel can exhibit practical and helpful values for clinical use.

### LncRNA-miRNA-mRNA network prediction and analyses

**Figure 7:**
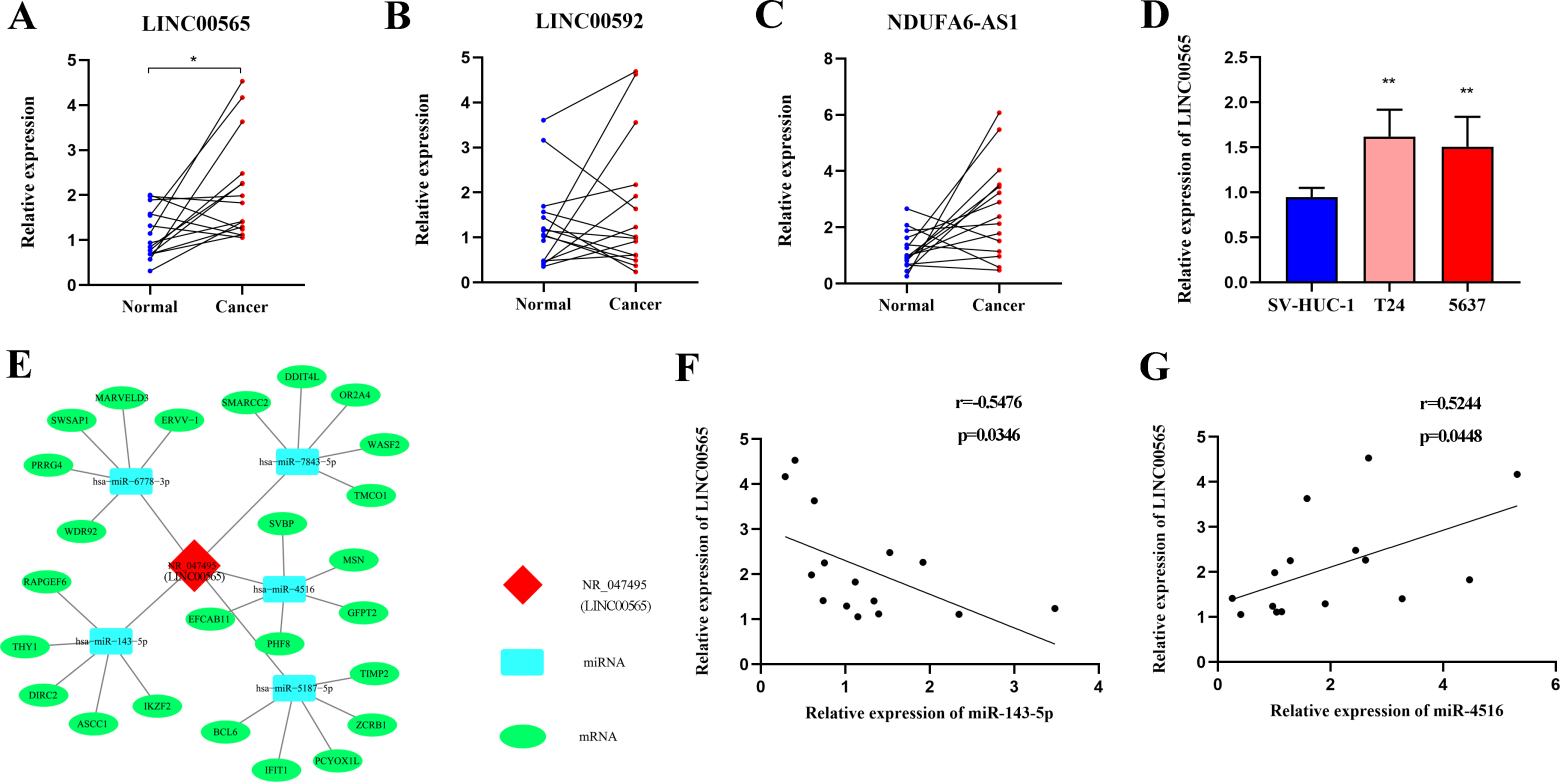
Expression analysis of LINC00565 and lncRNA-miRNA-mRNA network prediction. **(A-C)** Relative expression of LINC00565 **(A)**, LINC00592 **(B)**, and NDUFA6-AS1 **(C)** in tumor tissues of MIBC compared with adjacent noncancerous tissues. **(D)** Differential expression of LINC00565 in T24 and 5637 cells. **(E)** The candidate binding miRNAs and mRNAs to LINC00565. **(F, G)** The expression of miR-143-5p **(F)** and miR-4516 **(G)** showed significant correlation with levels of LINC00565 in MIBC **p<0.01, *p<0.05.

To investigate the biological functions of the three lncRNAs in pathomechanism, we measured their expression in 15 MIBC tissues and observed that LINC00565 was highly expressed in tumor tissues compared to adjacent noncancerous tissues (**Figures 7A–C**). In addition, LINC00565 overexpression was also detected in T24 and 5637 cells compared to SV-HUC-1 cells (**Figure 7D**). Subsequently, we constructed an lncRNA-miRNA-target gene network for LINC00565 using Cytoscape. The top five miRNAs that potentially bind to LINC00565 and the five most likely target genes for each miRNA were used to construct a network map (**Figure 7E**). For preliminary verification of the bioinformatics analysis, the expression correlation between LINC00565 and miRNAs was measured in the 15 MIBC tissues. Consistent with our prediction, the expression of miR-143-5p (Spearman’s correlation, r=-0.5476, *p*<0.05) was negatively correlated with the levels of LINC00565 in MIBC, and miR-4516 (Spearman’s correlation, r=0.5244, *p*<0.05) was positive correlated with the levels of LINC00565 (**Figures 7F, G**).

### Integrated analysis of three lncRNAs based on TCGA database

RNA sequencing data and overall survival (OS) information of 372 MIBC patients were downloaded from the Bladder Urothelial Carcinoma Project of TCGA (TCGA-BLCA Project). Kaplan-Meier analysis revealed that MIBC patients with high LINC00565 expression levels had significantly lower OS than patients with low expression levels (*p*=0.001, **Supplementary Figures S12A**). These findings further suggested that LINC00565 might have great potential to be used as promising biomarkers for MIBC. However, neither LINC00592 nor NDUFA6-AS1 influenced patients’ predicted survival (*p*=0.203 and *p*=0.051, **Supplementary Figures S12B, C**).

Considering chromosomes’ distribution of lncRNAs, copy number values of three lncRNA genes were measured in tissue samples of 15 MIBC patients and no variations were found (all *p* at>0.05, **Supplementary Figure S13**). In addition, relative data on the copy number values of three lncRNA genes were obtained from the Pan Cancer Atlas consisting of 375 BC samples and analysis revealed that copy number values of three lncRNA genes demonstrated no significant differences between NMIBC and MIBC (**Supplementary Figure S14**). These findings suggested that genomic imbalance may be not correlated with the dysregulation of these lncRNAs.

## Discussion

RC is the gold standard treatment for MIBC, whereas TUR are generally used for patients without muscular infiltration. Therefore, preoperative determination of muscle invasion is crucial for enhancing clinical decision-making. However, the current methods such as cystoscopy and imaging examinations, have certain deficiencies, including uncomfortable experience and limited accuracy. Consequently, non-invasive and precise biomarkers are required for MIBC diagnosis. In the present study, we analyzed the relevance of serum lncRNAs in MIBC using genome-wide HTS and RT-qPCR assays. A three-lncRNA panel was established using multivariate logistic regression analysis and demonstrated satisfactory diagnostic ability for MIBC. Furthermore, we found that the expression of LINC00565 was also upregulated in both BC tissues and cells and was significantly correlated with miR-143-5p and miR-4516. To our knowledge, this is the first study to demonstrate a potentially applicable lncRNA signature in serum that could aid in the development of improved muscle-invasive diagnosis.

Recently, circulating lncRNAs have attracted increasing attention as potential biomarkers for cancer based on their stable capacity in serum (24). Previous studies by us and others demonstrated that serum lncRNAs could be expressed in disease- or developmental-specific manners in various types of cancer, including BC (18–20, 25). However, these studies mostly focused on pre-selected signals or a limited number of lncRNAs, leaving the whole profile unknown, and may not be very reliable owing to the complicated pathogenesis of malignancy (26, 27). Thus, the present study was designed to comprehensively and systematically identify specific lncRNA profiles in serum of MIBC patients. Genome-wide analysis of serum lncRNAs was initially performed by HTS in patients with MIBC and NMIBC (28). Considering that the sequencing data may not be powerful enough to reflect the actual gene expression levels across individuals (29, 30), subsequent measurement of candidate lncRNAs in training and validation cohorts using RT-qPCR assays was performed (31). Finally, three lncRNAs were found to be differentially expressed in the MIBC-specific patterns. Using a multivariate logistic regression model, which has been shown to be more straightforward to implement and interpret (32), a 3-lncRNA panel was ultimately established. ROC analysis revealed that this panel can effectively discriminate patients with MIBC from those with NMIBC. Moreover, we found that with the pathological stage raising from T2 to T4, the diagnostic efficiency of the established signature was constantly increased, which could further support the specificity of the lncRNA signature for MIBC. These findings suggest that the 3-lncRNA panel based on comprehensive profile analysis has great potential to serve as effective noninvasive biomarkers for MIBC.

The inherent utility of lncRNA profiles in classifying MIBC has been investigated in many studies. Robertson et al. (33) reported that lncRNA expression clustering in tissues could identify distinct subsets of MIBC with differential EMT status, histological features, and patient survival. Song et al. (34) revealed a tissue-based 8-lncRNA signature as an independent prognostic indicator and classifier in different subgroups of MIBC. Moreover, lncRNAs exhibit great potential to provide additional information for higher-resolution subtyping of luminal-papillary MIBC (35). Nevertheless, investigations describing the characterization of circulating lncRNAs in MIBC are limited. Zhou et al. (17) highlighted the axis of LUCAT1/miR-199a/b-5p in MIBC pathogenesis and found that serum LUCAT1 was upregulated in MIBC. In the present study, we constructed an accurate diagnostic serum lncRNA panel for MIBC and tested it in patients diagnosed with BC. Patients deemed by this panel to have an elevated risk of MIBC development would then undergo further, typically invasive, and costly clinical workups. Furthermore, we established Fagan’s nomogram to facilitate the interpretation of panel results as useful information for clinicians (30, 36). With a supposed pre-test probability of 10%, the post-test probability of MIBC increased to 43% for a positive result and decreased to 3% for a negative result. Simple serum testing with diagnostic panel outcomes would be straightforward to implement and possibly serve as a cost-effective triaging tool, further benefiting at-risk populations. Nevertheless, as patients with MIBC in the present study were initially diagnosed, further researches are required to validate the diagnostic potential of serum lncRNAs for recurrent MIBC.

Research on the functional roles and molecular mechanisms of lncRNAs in tumor tissues and cells may contribute to the further evaluation of serum lncRNAs as biomarkers for cancer. It has been shown that the expression of lncRNAs could be upregulated in cancer cells not only to promote cancer cell proliferation and migration but also to increase secretion delivered by extracellular vesicles to the microenvironment (37). Among the three lncRNAs identified in our study, LINC00565 and LINC00592 have been reported to be involved in tumorigenesis and cancer development. In colorectal cancer, Shao et al. (38) reported that LINC00565 was mainly expressed in the cytoplasm and could stimulate the aggravation of cancer by upregulating EZH2. LINC00565 could also promote the progression of ovarian cancer by interacting with GAS6 (39). Yuan et al. (40) showed that LINC00592 is a potential cancer-related lncRNA in cervical cancer and may activate cancer progression through the regulation of transcription or structural integrity. In case of BC, LINC00592 was found to be located in the nucleus and could promote the growth and metastasis of cancer cells by enhancing the promoter methylation of WIF1 and decreasing WIF1 transcription (41). To characterize functions of lncRNAs in distinct subcellular compartments and reveal the physiopathological process they are involved in, further studies using techniques including APEX-RIP would be performed (42). In this study, analysis on the TCGA database revealed that MIBC patients with higher LINC00565 expression level had poorer prognosis. As TCGA data could be influenced by heterogeneity, irregularity, and other characteristics, it is possible that the values of LINC00592 nor NDUFA6-AS1 might be not fully utilized and further researches are needed to confirm our findings. Nevertheless, these findings indicated that the three lncRNAs may play important roles in cancer and have great potential to be investigated as indicators for cancer.

To further understand the functions of these differentially expressed lncRNAs, bioinformatic analysis was performed. We found some important functions or pathways that may explain the possible mechanisms underlying the increase in lncRNAs in MIBC. For instance, overexpressed lncRNAs are linked to taste transduction signaling, which might participate in the drug resistance of cancer (43). As lncRNAs could function as miRNA sponges or potent ceRNA molecules to regulate gene expression (12, 13), we predicted the potential miRNA targets of LINC00565 using TargetScan and Miranda databases, and Spearman’s correlation analysis revealed that LINC00565 displayed a tendency of negative correlation with miR-143-5p but a positive correlation with miR-4516 in MIBC. The association of miRNAs with cancer indicates that LINC00565 may play a regulatory role in the development of MIBC. For instance, downregulated miR-143 was reported in the serum of BC (44) and could modulate apoptosis and malignant phenotype of BC cells via PCMT1 (45). In addition, miR-4516 was identified as a novel oncogene by targeting PTPN14 in glioblastoma (46), and stromal loss of miR-4516 could promote FOSL1-dependent proliferation and malignancy of triple-negative breast cancer (47). Additional studies are required to verify the target genes of the three lncRNAs and elucidate the underlying mechanisms that regulate their functions.

Among the three lncRNAs, LINC00565 was transcribed from intergenic regions. Moreover, we found that the most up-regulated lncRNAs were located in chromosome 2 and transcribed from intergenic regions in unannotated sequences of gene. These findings highlighted the important roles of chromosome location and source of the lncRNA formation in cancer (48, 49). Further researches focusing on the findings based on the different expression patterns of lncRNAs in chromosomes and transcription should be performed to elucidate the biological roles of lncRNAs in MIBC. Moreover, the data obtained from the Pan Cancer Atlas demonstrated that copy numbers of the three lncRNAs were similar between NMIBC and MIBC, and it is possible that other regulating mechanisms such as DNA methylation and histone modification, may exist in the abnormal expression of lncRNAs in MIBC (50, 51). Additional studies are required to elucidate the underlying mechanisms that regulate their expression and functions.

Despite these promising results, the present study had several limitations. First, more sensitive technologies such as ddPCR should be performed to validate the results by RT-qPCR, as ddPCR could show better performance for low nucleic acid load samples and reduce the production of false positive reports (52). Second, the sample size is not large enough and the capacity of the lncRNA panel to discriminate MIBC from other types of invasive tumors is still unknown. Further large-scale and multi-center studies including more females are needed to validate the diagnostic efficiency. Third, despite previous speculation declaring the possible mechanisms of stably detected lncRNA in serum included binding with protein and folding into complex secondary and tertiary structures (53, 54), the source and secretion mechanisms of serum lncRNA remain unclear. Moreover, the precise biological functions of the identified lncRNAs have not yet been clarified. More intensive studies are needed to illustrate the molecular mechanisms of lncRNAs involved in MIBC and to make these results more convincing for future clinical applications.

In conclusion, we defined a distinctive serum 3-lncRNA signature for the diagnosis of MIBC. Although further studies are needed to confirm the results of this study, our findings highlight the clinical value of serum lncRNAs in the diagnosis of MIBC and provide potential directions for exploring the roles of lncRNAs in cancer pathogenesis.

## Data Availability

The original contributions presented in the study are included in the article/**Supplementary Material**. Further inquiries can be directed to the corresponding authors.

## References

[1] Sung H Ferlay J Siegel RL Laversanne M Soerjomataram I Jemal A. Global cancer statistics 2020: GLOBOCAN estimates of incidence and mortality worldwide for 36 cancers in 185 countries. CA Cancer J Clin. (2021) 71:209–49. doi: 10.3322/caac.21660 33538338

[2] Funt SA Rosenberg JE. Systemic, perioperative management of muscle-invasive bladder cancer and future horizons. Nat Rev Clin Oncol. (2017) 14:221–34. doi: 10.1038/nrclinonc.2016.188 PMC6054138 27874062

[3] Tan TZ Rouanne M Tan KT Huang RY Thiery JP. Molecular subtypes of urothelial bladder cancer: results from a meta-cohort analysis of 2411 tumors. Eur Urol. (2019) 75:423–32. doi: 10.1016/j.eururo.2018.08.027 30213523

[4] Alfred Witjes J Lebret T Compérat EM Cowan NC De Santis M Bruins HM. Updated 2016 EAU guidelines on muscle-invasive and metastatic bladder cancer. Eur Urol. (2017) 71:462–75. doi: 10.1016/j.eururo.2016.06.020 27375033

[5] Patel VG Oh WK Galsky MD. Treatment of muscle-invasive and advanced bladder cancer in 2020. CA Cancer J Clin. (2020) 70:404–23. doi: 10.3322/caac.21631 32767764

[6] Yang Y Zou X Wang Y Ma X. Application of deep learning as a noninvasive tool to differentiate muscle-invasive bladder cancer and non-muscle-invasive bladder cancer with CT. Eur J Radiol. (2021) 139:109666. doi: 10.1016/j.ejrad.2021.109666 33798819

[7] Hollenbeck BK Miller DC Dunn RL Montie JE Wei JT. The effects of stage divergence on survival after radical cystectomy for urothelial cancer. Urol Oncol. (2005) 23:77–81. doi: 10.1016/j.urolonc.2004.08.012 15869990

[8] Chang SS Hassan JM Cookson MS Wells N Smith JA Jr. Delaying radical cystectomy for muscle invasive bladder cancer results in worse pathological stage. J Urol. (2003) 170:1085–7. doi: 10.1097/01.ju.0000086828.26001.ca 14501697

[9] McKibben MJ Woods ME. Preoperative imaging for staging bladder cancer. Curr Urol Rep. (2015) 16:22. doi: 10.1007/s11934-015-0496-8 25724433

[10] Ng K Stenzl A Sharma A Vasdev N. Urinary biomarkers in bladder cancer: A review of the current landscape and future directions. Urol Oncol. (2021) 39:41–51. doi: 10.1016/j.urolonc.2020.08.016 32919875

[11] Ouyang J Zhong Y Zhang Y Yang L Wu P Hou X. Long non-coding RNAs are involved in alternative splicing and promote cancer progression. Br J Cancer. (2022) 126:1113–24. doi: 10.1038/s41416-021-01600-w PMC9023592 34750493

[12] Xue ST Zheng B Cao SQ Ding JC Hu GS Liu W. Long Non-coding RNA LINC00680 Functions as a ceRNA to Promote Esophageal Squamous Cell Carcinoma Progression Through the miR-423-5p/PAK6 axis. Mol Cancer. (2022) 21:69. doi: 10.1186/s12943-022-01539-3 35255921 PMC8900330

[13] Xu TP Ma P Wang WY Shuai Y Wang YF Yu T. KLF5 and MYC modulated LINC00346 contributes to gastric cancer progression through acting as a competing endogeous RNA and indicates poor outcome. Cell Death Differ. (2019) 26:2179–93. doi: 10.1038/s41418-018-0236-y PMC6888833 30770877

[14] Fu P Gong B Li H Luo Q Huang Z Shan R. Combined identification of three lncRNAs in serum as effective diagnostic and prognostic biomarkers for hepatitis B virus-related hepatocellular carcinoma. Int J Cancer. (2022) 151:1824–34. doi: 10.1002/ijc.34201 35802466

[15] Qi P Zhou XY Du X. Circulating long non-coding RNAs in cancer: current status and future perspectives. Mol Cancer. (2016) 15:39. doi: 10.1186/s12943-016-0524-4 27189224 PMC4869386

[16] Tan SK Pastori C Penas C Komotar RJ Ivan ME Wahlestedt C. Serum long noncoding RNA HOTAIR as a novel diagnostic and prognostic biomarker in glioblastoma multiforme. Mol Cancer. (2018) 17:74. doi: 10.1186/s12943-018-0822-0 29558959 PMC5861620

[17] Zhou Y Song X Li X Li H Peng Y. Serum LUCAT1 Implicates the Pathogenesis of Muscle-invasive Bladder Cancer via Targeting miR-199a-5p and miR-199b-5p. J Mol Histol. (2020) 51:583–91. doi: 10.1007/s10735-020-09907-3 32844284

[18] Xie Y Zhang Y Du L Jiang X Yan S Duan W. Circulating long noncoding RNA act as potential novel biomarkers for diagnosis and prognosis of non-small cell lung cancer. Mol Oncol. (2018) 12:648–58. doi: 10.1002/1878-0261.12188 PMC5928376 29504701

[19] Zhang S Du L Wang L Jiang X Zhan Y Li J. Evaluation of serum exosomal lncRNA-based biomarker panel for diagnosis and recurrence prediction of bladder cancer. J Cell Mol Med. (2019) 23:1396–405. doi: 10.1111/jcmm.14042 PMC6349164 30467945

[20] Wang R Du L Yang X Jiang X Duan W Yan S. Identification of long noncoding RNAs as potential novel diagnosis and prognosis biomarkers in colorectal cancer. J Cancer Res Clin Oncol. (2016) 142:2291–301. doi: 10.1007/s00432-016-2238-9 PMC11819400 27591862

[21] Yang Y Qu A Wu Q Zhang X Wang L Li C. Prognostic value of a hypoxia-related microRNA signature in patients with colorectal cancer. Aging (Albany NY). (2020) 12:35–52. doi: 10.18632/aging.102228 31926112 PMC6977676

[22] Liu H Bi J Dong W Yang M Shi J Jiang N. Invasion-related circular RNA circFNDC3B inhibits bladder cancer progression through the miR-1178-3p/G3BP2/SRC/FAK axis. Mol Cancer. (2018) 17:161. doi: 10.1186/s12943-018-0908-8 30458784 PMC6245936

[23] Siegel RL Miller KD Fuchs HE Jemal A. Cancer statistics, 2022. CA Cancer J Clin. (2022) 72:7–33. doi: 10.3322/caac.21708 35020204

[24] Chandra Gupta S Nandan Tripathi Y. Potential of long non-coding RNAs in cancer patients: from biomarkers to therapeutic targets. Int J Cancer. (2017) 140:1955–67. doi: 10.1002/ijc.30546 27925173

[25] Zhang D Du D Yi S Li X. LncRNA PCAT6: A potential biomarker for diagnosis and prognosis of bladder cancer. Ann Diagn Pathol. (2020) 49:151642. doi: 10.1016/j.anndiagpath.2020.151642 33142195

[26] Yan S Du L Jiang X Duan W Li J Xie Y. Evaluation of serum exosomal lncRNAs as diagnostic and prognostic biomarkers for esophageal squamous cell carcinoma. Cancer Manag Res. (2020) 12:9753–63. doi: 10.2147/CMAR.S250971 PMC7548224 33116835

[27] Dang Q Liu Z Liu Y Wang W Yuan W Sun Z. LncRNA profiles from notch signaling: implications for clinical management and tumor microenvironment of colorectal cancer. Front Immunol. (2022) 13:953405. doi: 10.3389/fimmu.2022.953405 35958606 PMC9359081

[28] Zheng W Chen C Chen S Fan C Ruan H. Integrated analysis of long non-coding RNAs and mRNAs associated with peritendinous fibrosis. J Adv Res. (2018) 15:49–58. doi: 10.1016/j.jare.2018.08.001 30581612 PMC6300459

[29] Sun J Jiang R Song M Yao J Hou S Zhu Y. Pathological Grade-Associated Transcriptome Profiling of lncRNAs and mRNAs in Gliomas. Front Oncol. (2020) 10):253. doi: 10.3389/fonc.2020.00253 32211318 PMC7076085

[30] Qu A Wang W Yang Y Zhang X Dong Y Zheng G. A serum piRNA signature as promising non-invasive diagnostic and prognostic biomarkers for colorectal cancer. Cancer Manag Res. (2019) 11:3703–20. doi: 10.2147/CMAR.S193266 PMC6500438 31118791

[31] Du L Duan W Jiang X Zhao L Li J Wang R. Cell-free lncRNA expression signatures in urine serve as novel non-invasive biomarkers for diagnosis and recurrence prediction of bladder cancer. J Cell Mol Med. (2018) 22:2838–45. doi: 10.1111/jcmm.13578 PMC5908122 29516641

[32] Blyuss O Zaikin A Cherepanova V Munblit D Kiseleva EM Prytomanova OM. Development of pancRISK, a urine biomarker-based risk score for stratified screening of pancreatic cancer patients. Br J Cancer. (2020) 122:692–6. doi: 10.1038/s41416-019-0694-0 PMC7054390 31857725

[33] Robertson AG Kim J Al-Ahmadie H Bellmunt J Guo G Cherniack AD. Comprehensive molecular characterization of muscle-invasive bladder cancer. Cell. (2018) 174:1033. doi: 10.1016/j.cell.2017.09.007 30096301 PMC6297116

[34] Song Y Jin D Chen J Luo Z Chen G Yang Y. Identification of an immune-related long non-coding RNA signature and nomogram as prognostic target for muscle-invasive bladder cancer. Aging (Albany NY). (2020) 12:12051–73. doi: 10.18632/aging.103369 PMC7343518 32579540

[35] de Jong JJ Liu Y Robertson AG Seiler R Groeneveld CS van der Heijden MS. Long non-coding RNAs identify a subset of luminal muscle-invasive bladder cancer patients with favorable prognosis. Genome Med. (2019) 11:60. doi: 10.1186/s13073-019-0669-z 31619281 PMC6796434

[36] Fagan TJ. Letter: nomogram for bayes theorem. N Engl J Med. (1975) 293:257. doi: 10.1056/NEJM197507312930513 1143310

[37] Hu W Liu C Bi ZY Zhou Q Zhang H Li LL. Comprehensive landscape of extracellular vesicle-derived RNAs in cancer initiation, progression, metastasis and cancer immunology. Mol Cancer. (2020) 19:102. doi: 10.1186/s12943-020-01199-1 32503543 PMC7273667

[38] Shao X Zhao T Xi L Zhang Y He J Zeng J. LINC00565 promotes the progression of colorectal cancer by upregulating EZH2. Oncol Lett. (2021) 21:53. doi: 10.3892/ol.2020.12314 33281964 PMC7709565

[39] Gong M Luo C Meng H Li S Nie S Jiang Y. Upregulated LINC00565 accelerates ovarian cancer progression by targeting GAS6. Onco Targets Ther. (2019) 12:10011–22. doi: 10.2147/OTT.S227758 PMC6875503 31819497

[40] Yuan LY Qin X Li L Zhou J Zhou M Li X. The transcriptome profiles and methylation status revealed the potential cancer-related lncRNAs in patients with cervical cancer. J Cell Physiol. (2019) 234:9756–63. doi: 10.1002/jcp.27661 30362566

[41] Wu T Li N Wu X Du Y Tang Z. LncRNA LINC00592 mediates the promoter methylation of WIF1 to promote the development of bladder cancer. Open Med (Wars). (2023) 18:20230788. doi: 10.1515/med-2023-0788 37786775 PMC10541805

[42] Kaewsapsak P Shechner DM Mallard W Rinn JL Ting AY. Live-cell mapping of organelle-associated RNAs via proximity biotinylation combined with protein-RNA crosslinking. Elife. (2017) 6:e29224. doi: 10.7554/eLife.29224 29239719 PMC5730372

[43] Costa AR Duarte AC Costa-Brito AR Gonçalves I Santos CRA. Bitter taste signaling in cancer. Life Sci. (2023) 315:121363. doi: 10.1016/j.lfs.2022.121363 36610638

[44] Wen Z Huang G Lai Y Xiao L Peng X Liu K. Diagnostic Panel of Serum miR-125b-5p, miR-182-5p, and miR-200c-3p as Non-invasive Biomarkers for Urothelial Bladder Cancer. Clin Transl Oncol. (2022) 24:909–18. doi: 10.1007/s12094-021-02741-3 35028929

[45] Dong LM Zhang XL Mao MH Li YP Zhang XY Xue DW. LINC00511/miRNA-143-3p modulates apoptosis and Malignant phenotype of bladder carcinoma cells via PCMT1. Front Cell Dev Biol. (2021) 9:650999. doi: 10.3389/fcell.2021.650999 33898446 PMC8063617

[46] Cui T Bell EH McElroy J Becker AP Gulati PM Geurts M. miR-4516 predicts poor prognosis and functions as a novel oncogene via targeting PTPN14 in human glioblastoma. Oncogene. (2019) 38:2923–36. doi: 10.1038/s41388-018-0601-9 PMC6497411 30559405

[47] Kim JE Kim BG Jang Y Kang S Lee JH Cho NH. The stromal loss of miR-4516 promotes the FOSL1-dependent proliferation and Malignancy of triple negative breast cancer. Cancer Lett. (2020) 469:256–65. doi: 10.1016/j.canlet.2019.10.039 31672492

[48] Ransohof JD Wei Y Khavari PA. The functions and unique features of long intergenic non-coding RNA. Nat Rev Mol Cell Biol. (2018) 19:143–57. doi: 10.1038/nrm.2017.104 PMC5889127 29138516

[49] Heskett MB Vouzas AE Smith LG Yates PA Boniface C Bouhassira EE. Epigenetic control of chromosome-associated lncRNA genes essential for replication and stability. Nat Commun. (2022) 13:6301. doi: 10.1038/s41467-022-34099-7 36273230 PMC9588035

[50] Hadji F Boulanger MC Guay SP Gaudreault N Amellah S Mkannez G. Altered DNA methylation of long noncoding RNA H19 in calcific aortic valve disease promotes mineralization by silencing NOTCH1. Circulation. (2016) 134:1848–62. doi: 10.1161/CIRCULATIONAHA.116.023116 27789555

[51] Zhang E Han L Yin D He X Hong L Si X. H3K27 acetylation activated-long non-coding RNA CCAT1 affects cell proliferation and migration by regulating SPRY4 and HOXB13 expression in esophageal squamous cell carcinoma. Nucleic Acids Res. (2017) 45:3086–101. doi: 10.1093/nar/gkw1247 PMC5389582 27956498

[52] Liu X Feng J Zhang Q Guo D Zhang L Suo T. Analytical comparisons of SARS-COV-2 detection by qRT-PCR and ddPCR with multiple primer/probe sets. Emerg Microbes Infect. (2020) 9:1175–79. doi: 10.1080/22221751.2020.1772679 PMC7448863 32448084

[53] Shi T Gao G Cao Y. Long noncoding RNAs as novel biomarkers have a promising future in cancer diagnostics. Dis Markers. (2016) 2016:9085195. doi: 10.1155/2016/9085195 27143813 PMC4842029

[54] Hewson C Morris KV. Form and function of exosome-associated long non-coding RNAs in cancer. Curr Top Microbiol Immunol. (2016) 394:41–56. doi: 10.1007/82_2015_486 26739961

