# Supplementary material for: RNA-seq profiling identified a three-lncRNA panel in serum as potential biomarker for muscle-invasive bladder cancer
Source: Front Oncol. 2024 Dec 16;14:1451009. doi: 10.3389/fonc.2024.1451009 (PMC11683095; doi:10.3389/fonc.2024.1451009)
Supplement: Supplementary file 1 [file DataSheet1.docx]

**SUPPLEMENTARY FILES**

**SUPPLEMENTARY METHOD**

**QPCR Assays for Gene Copy Number Analysis**

gDNA was isolated from tissues using FastPure Blood/Cell/Tissue/Bacteria DNA Isolation Mini Kit (Vazyme, Nanjing, China). TaqMan^TM^ Copy Number Assays (Applied Biosystems, USA) was used to assess gene copy number. qPCR reactions were performed on StepOne Plus PCR instrument (Applied Biosystems, USA) using with TaqMan Genotyping PCR Master Mix. RNaseP was used as the endogenous controls. Relative quantification was conducted by CopyCaller Software.

**SUPPLEMENTARY FIGURES**

**
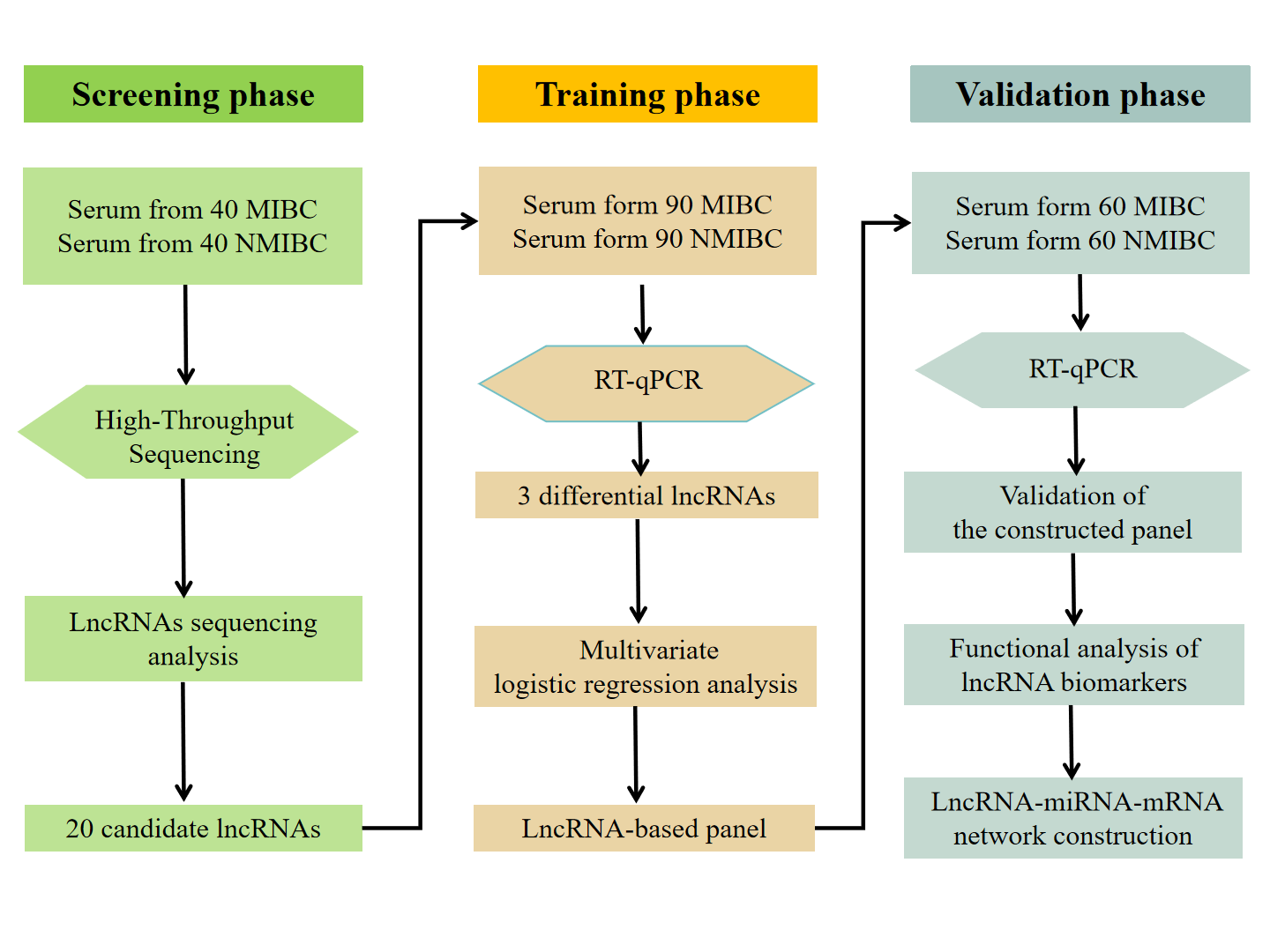
**

**Supplementary Figure S1**. Study flowchart.


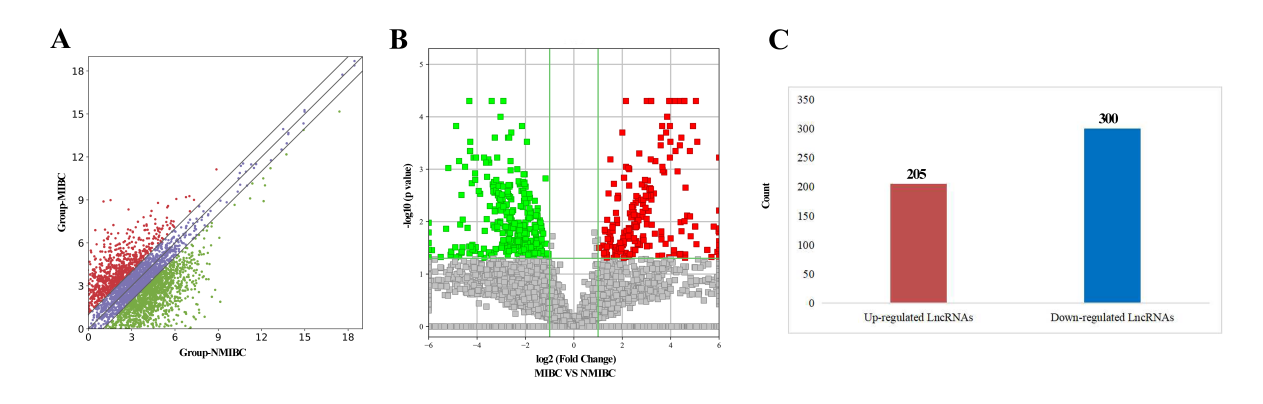


**Supplementary Figure S2**. Analysis of lncRNAs in MIBC by RNA-sequencing. (A) The scatter plot demonstrated the expression profiles of lncRNAs between MIBC and NMIBC. Red points showed upregulated lncRNAs with FC larger than 2 and green points indicated downregulated lncRNAs. (B) The volcano plot represented expression of lncRNAs between the two groups. Vertical green lines correspond to a 2.0-fold (log2 scaled) upregulated or downregulated changes, while horizontal green line marks a *p*-value of 0.05 (–log10 scaled). Red and green points in the plot refer to differently expressed lncRNAs. (C) The amount of differently expressed lncRNAs in MIBC.

**
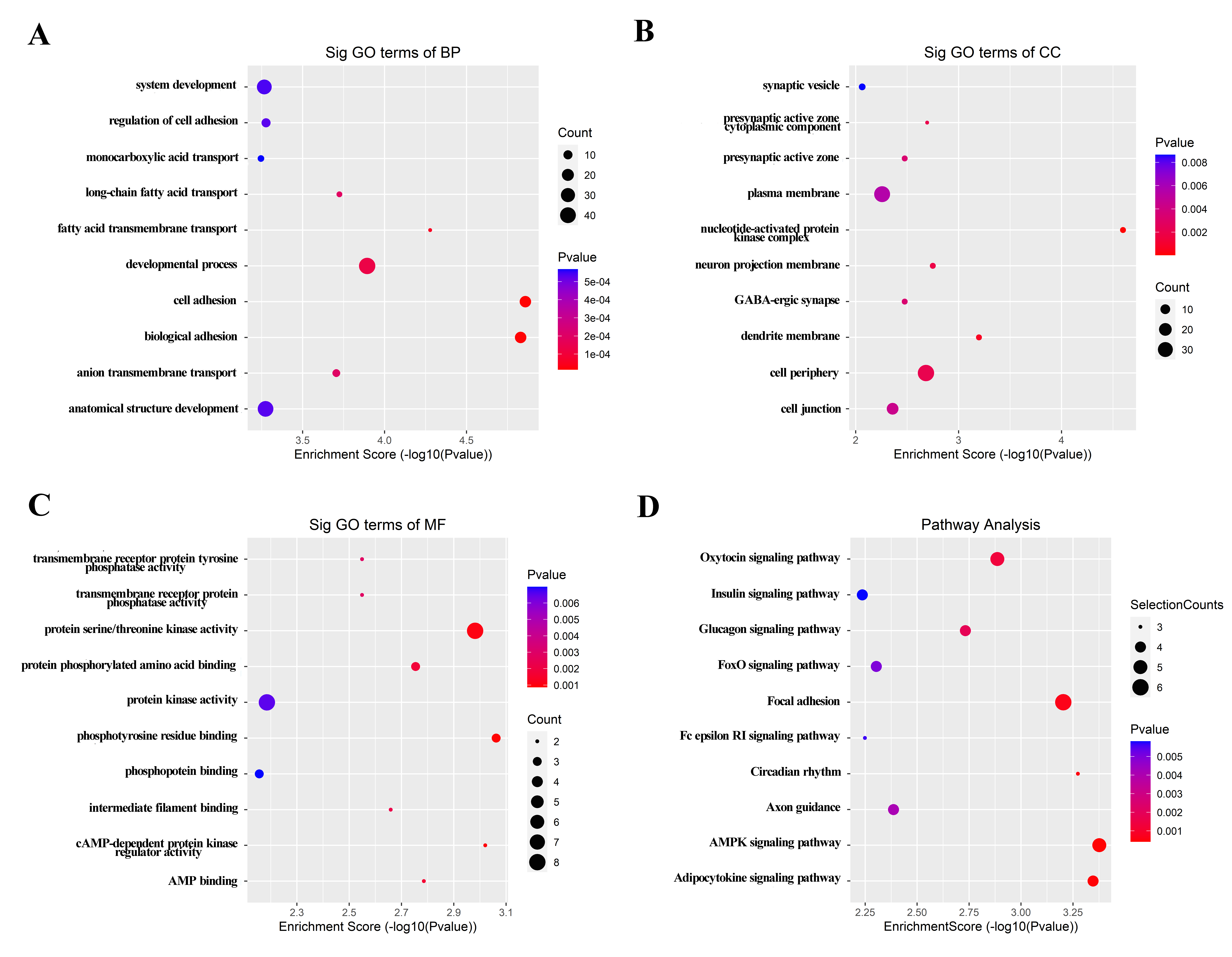
**

**Supplementary Figure S3**. GO and KEGG analysis of downexpressed lncRNAs in serum of MIBC.

**
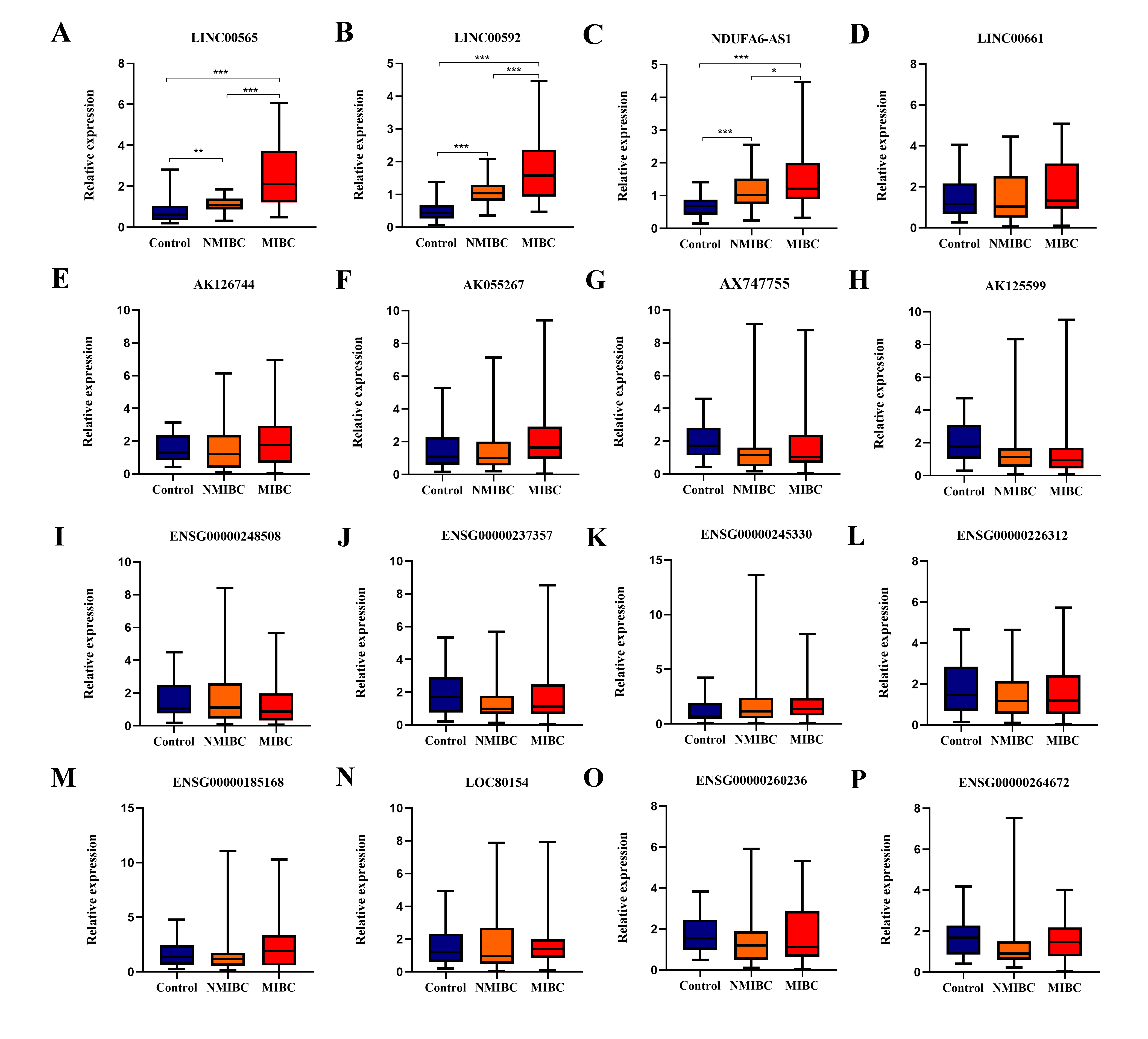
**

**Supplementary Figure S4**. Expression patterns of candidate lncRNAs revealed by HTS in patients with MIBC (n=30), NMIBC (n=30) and healthy controls (n=30) using RT-qPCR in the training set, ****p*<0.001,***p*<0.01, **p*<0.05.

**
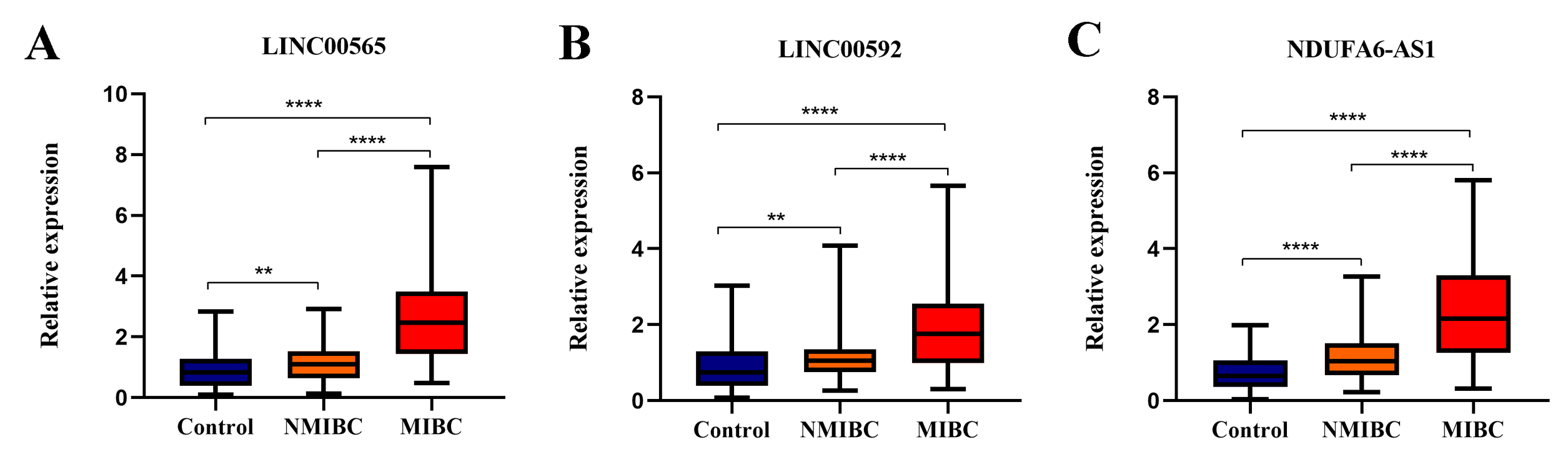
**

**Supplementary Figure S5**. The differential expression patterns of LINC00565 (A), LINC00592(B), and NDUFA6-AS1(C) in MIBC (n=90) compared with healthy controls (n=90) in the training set. *****p*<0.0001,***p*<0.01.


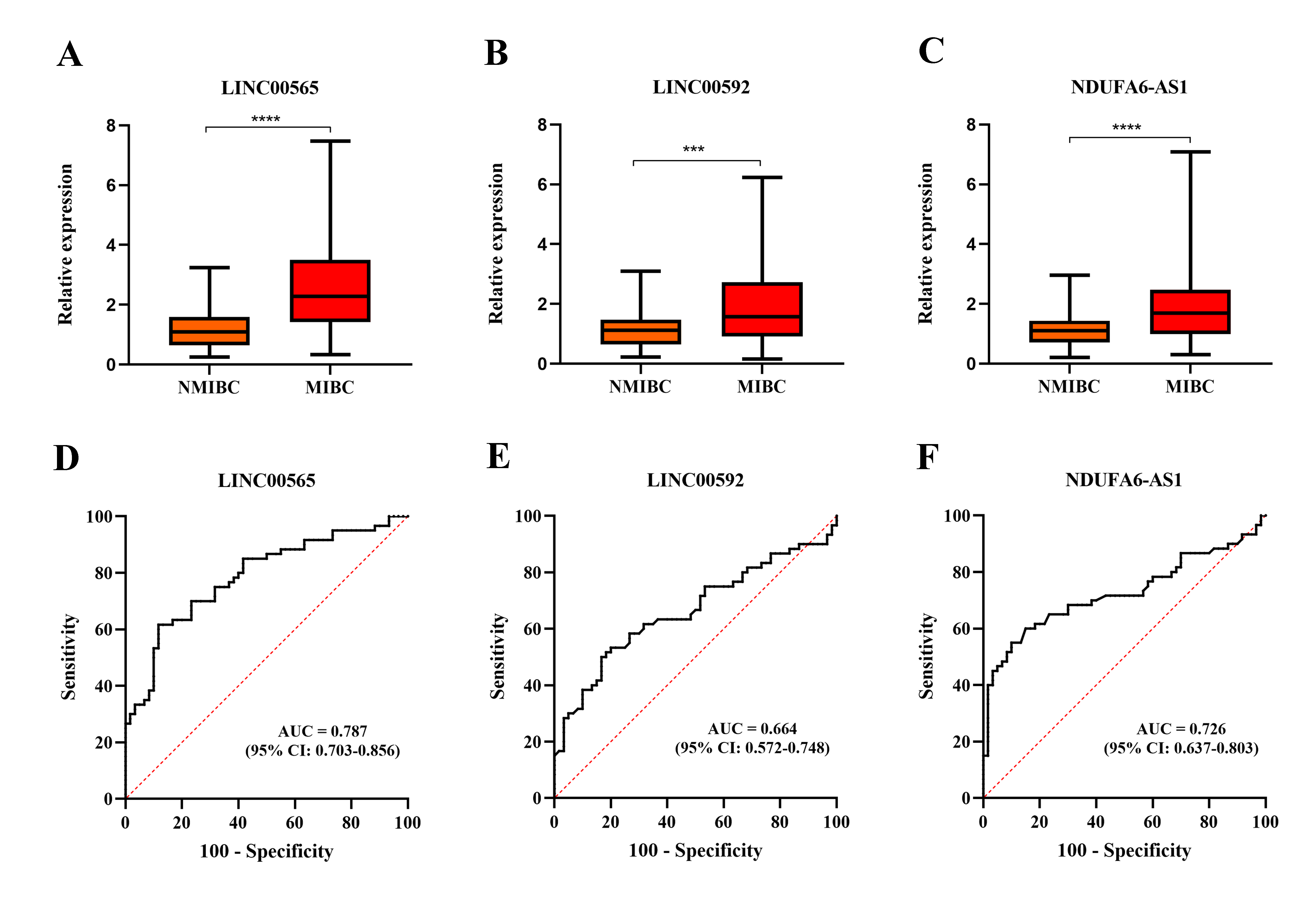


**Supplementary Figure S6**. The Box-whisker plots and ROC plots represent for LINC00565, LINC00592, and NDUFA6-AS1 in the validation set. (A-C) Differential expression patterns of LINC00565(A), LINC00592(B), and NDUFA6-AS1(C) among MIBC patients and NMIBC patients using RT-qPCR assay. (D-F) ROC curve analysis for diagnosis of MIBC using LINC00565(D), LINC00592(E), and NDUFA6-AS1(F), *****p*<0.0001,****p*<0.001.


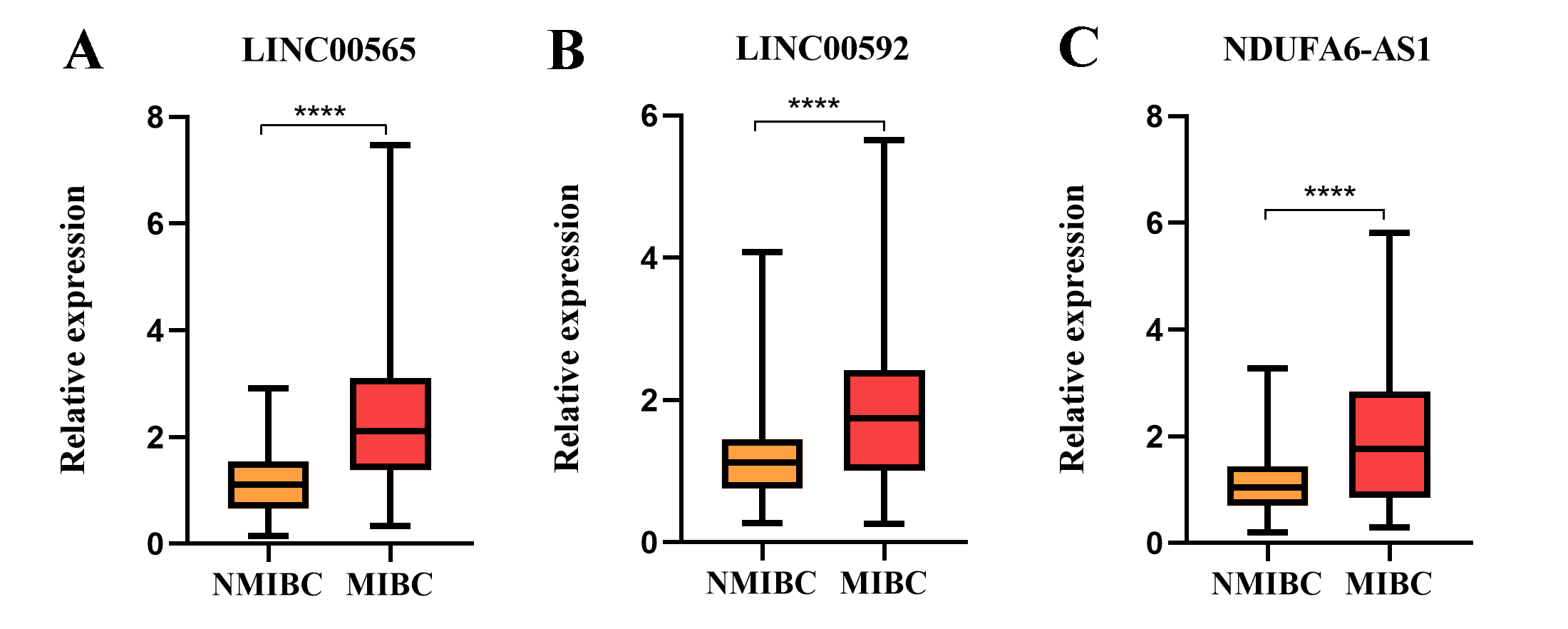


**Supplementary Figure S7**. Differential expression patterns of LINC00565(A), LINC00592(B) and NDUFA6-AS1(C) between NMIBC and MIBC in the cohort including 98 females and 98 males, *****p*<0.0001.


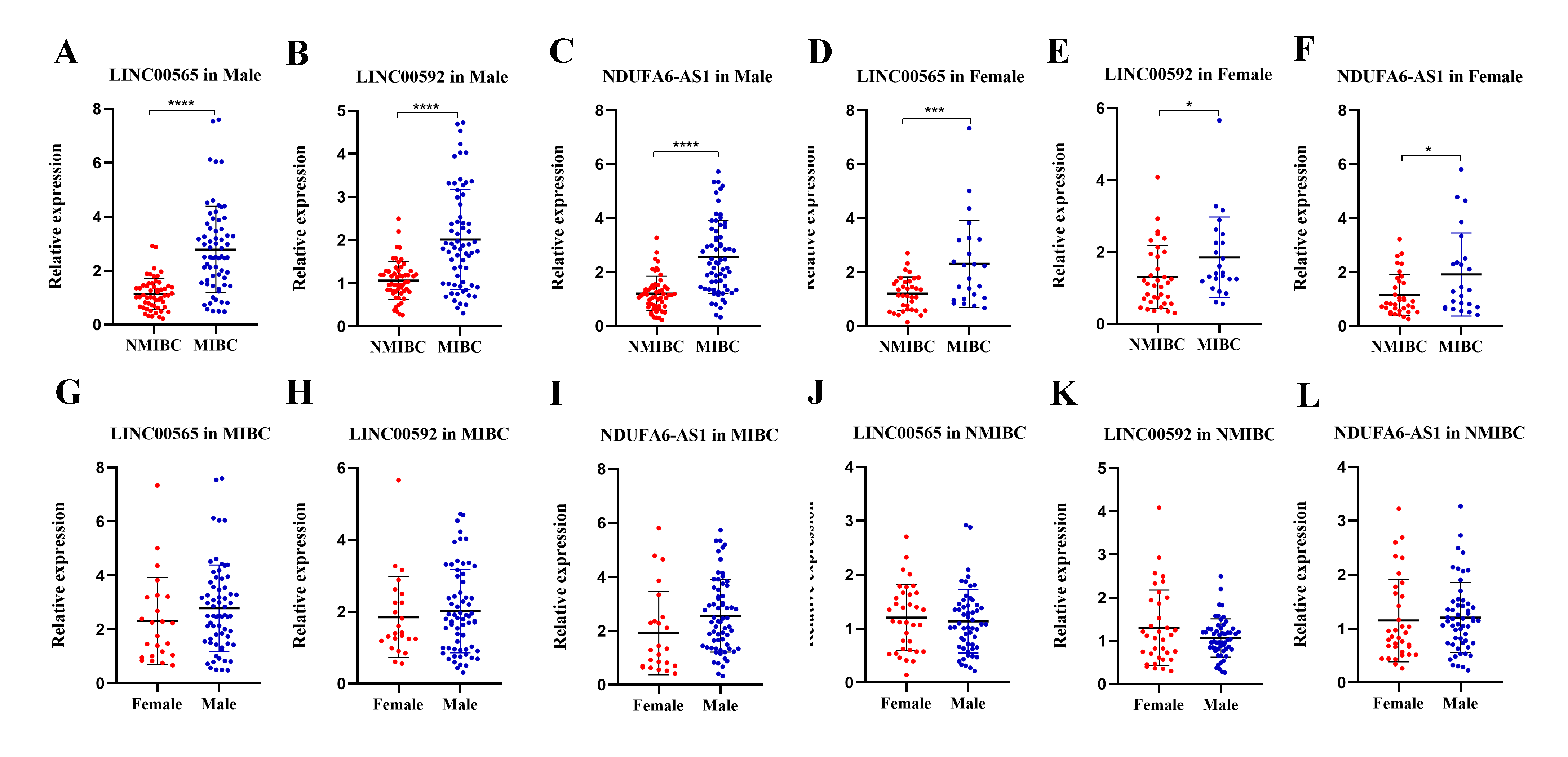


**Supplementary Figure S8**. Analysis on expression patterns of three lncRNAs based on sex in the training set. (A-C) Differential expression of LINC00565(A), LINC00592(B) and NDUFA6-AS1(C) between NMIBC and MIBC in male. (D-F) Differential expression of LINC00565(D), LINC00592(E) and NDUFA6-AS1(F) between NMIBC and MIBC in female. (G-I) Similar expression of LINC00565(G), LINC00592(H) and NDUFA6-AS1(I) between female and male in MIBC. (J-L) Similar expression of LINC00565(J), LINC00592(K) and NDUFA6-AS1(L) between female and male in NMIBC. *****p*<0.0001, ****p*<0.001, **p*<0.05.


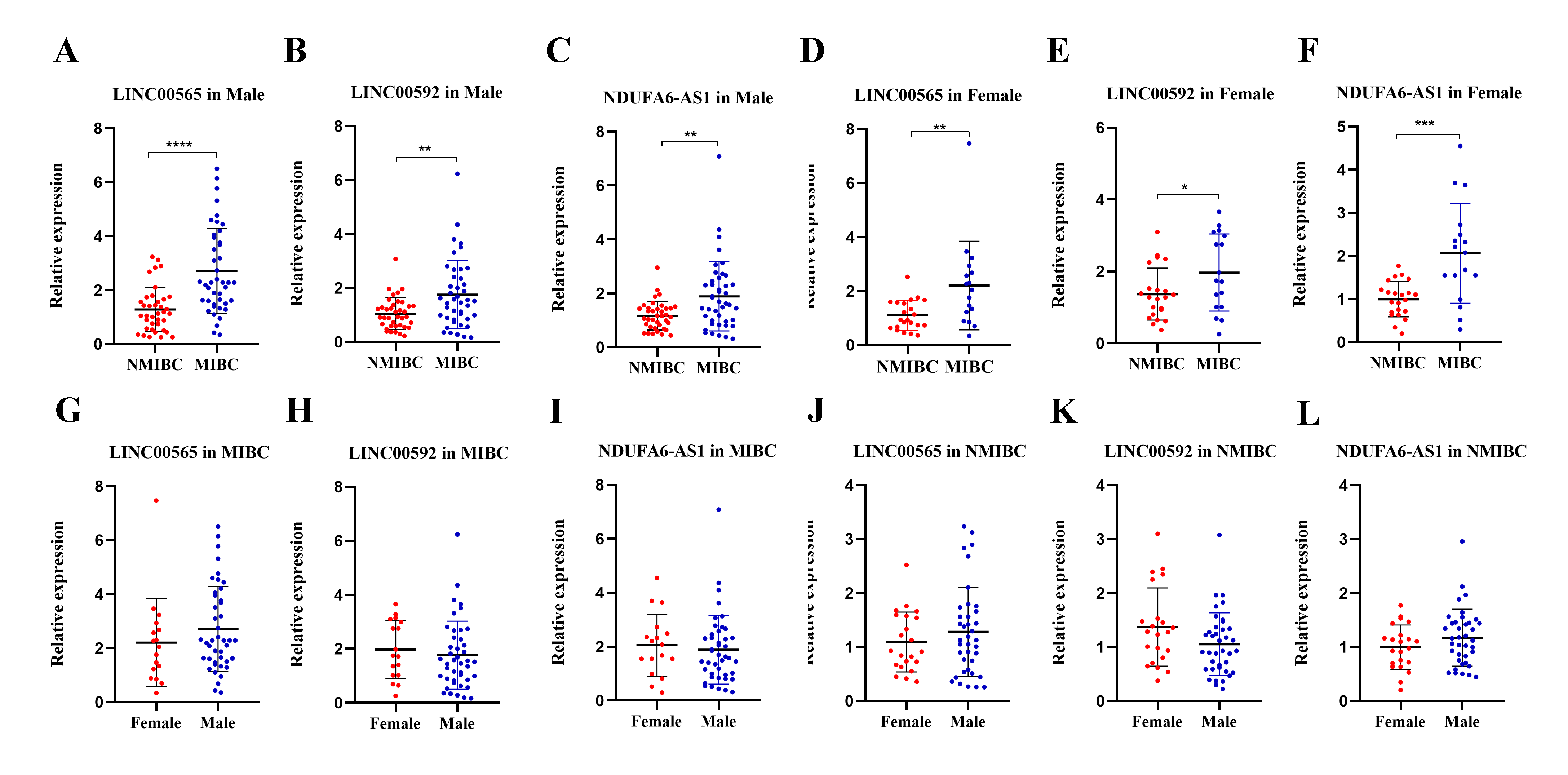


**Supplementary Figure S9**. Analysis on expression patterns of three lncRNAs based on sex in the validation set. (A-C) Differential expression of LINC00565(A), LINC00592(B) and NDUFA6-AS1(C) between NMIBC and MIBC in male. (D-F) Differential expression of LINC00565(D), LINC00592(E) and NDUFA6-AS1(F) between NMIBC and MIBC in female. (G-I) Similar expression of LINC00565(G), LINC00592(H) and NDUFA6-AS1(I) between female and male in MIBC. (J-L) Similar expression of LINC00565(J), LINC00592(K) and NDUFA6-AS1(L) between female and male in NMIBC. *****p*<0.0001, ****p*<0.001, ***p*<0.01, **p*<0.05.


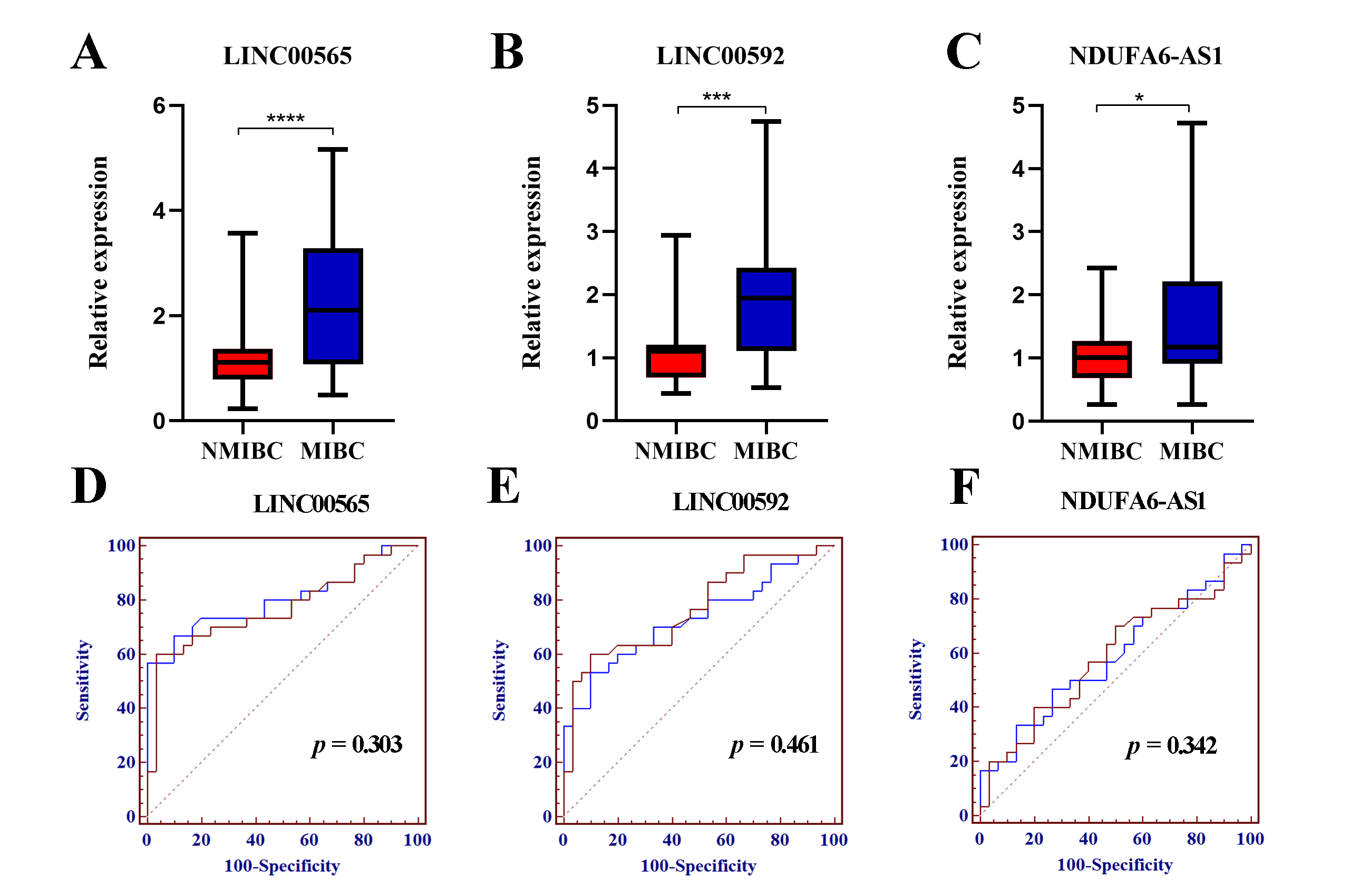


**Supplementary Figure S10**. Expression of three lncRNAs revealed by RT-qPCR based on RNA concentration measured by Qubit. (A-C) Differential expression of LINC00565(A), LINC00592(B), and NDUFA6-AS1(C) in MIBC using RT-qPCR based on Qubit. (D-F) Comparison of AUCs of LINC00565(D), LINC00592(E), and NDUFA6-AS1(F) for MIBC between RT-qPCR assays based on Qubit and NanoDrop. The brown line and blue line respectively represent the ROC curves using RT-qPCR based on Qubit and NanoDrop. *****p*<0.0001, ****p*<0.001, **p*<0.05.


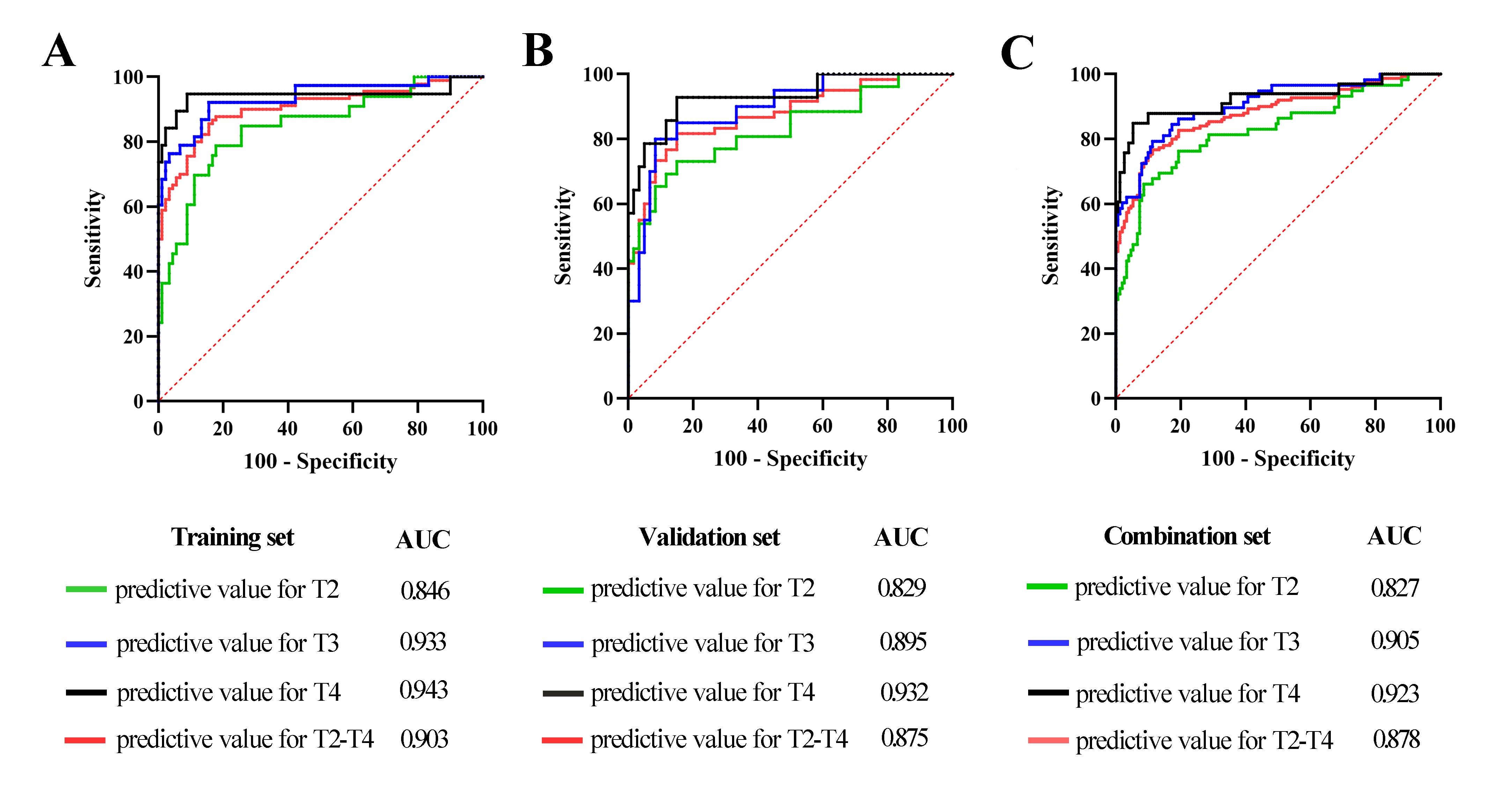


**Supplementary Figure S11.** ROC curve analysis for the 3-lnRNA panel in the training set (A), validation set (B) and combination set (C) in different stages of MIBC.


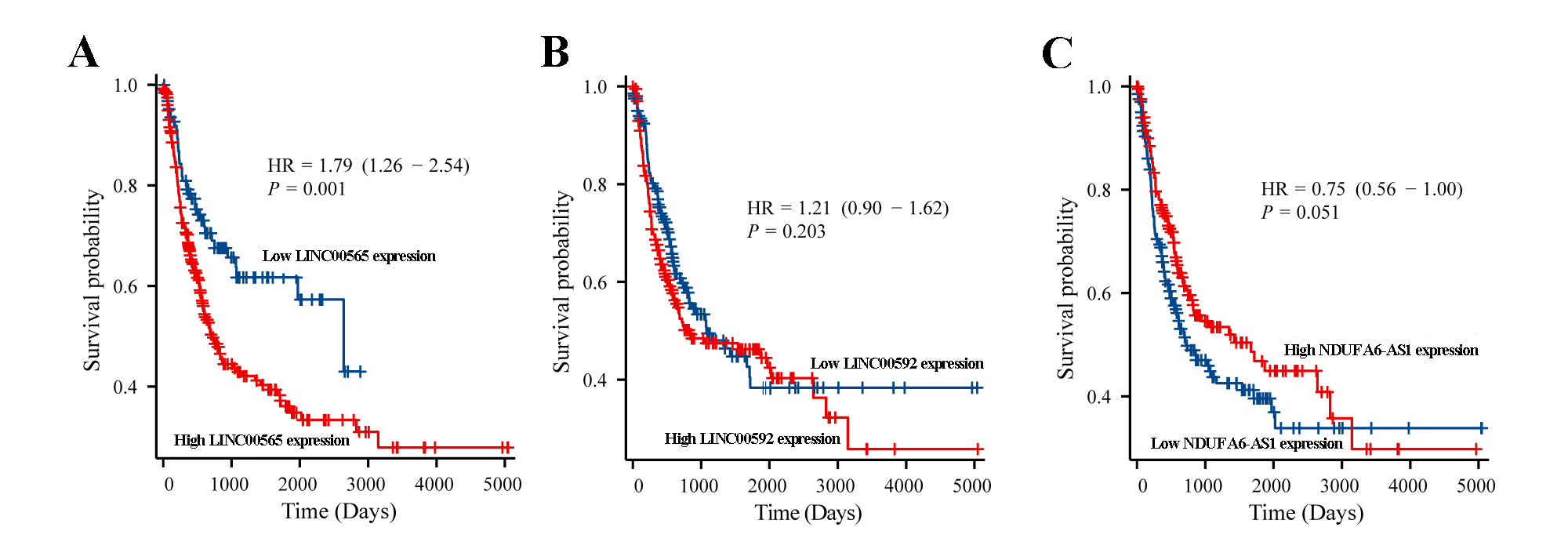


**Supplementary Figure S12**. Kaplan-Meier curves for overall survival of MIBC patients according to low and high expression levels of LINC00565 (A), LINC00592 (B) and NDUFA6-AS1(C) in the TCGA database.


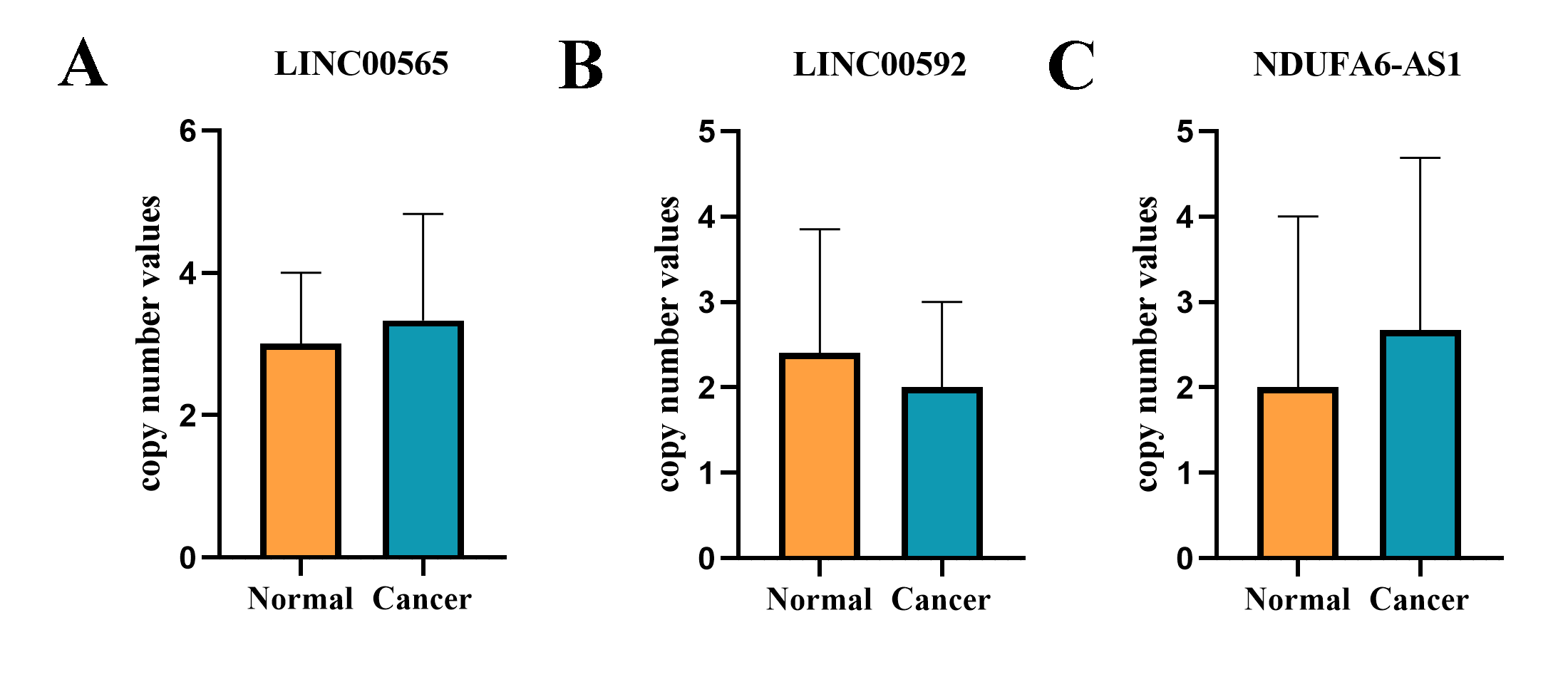


**Supplementary Figure S13**. Comparisons of estimated copy numbers of LINC00565(A), LINC00592(B), and NDUFA6-AS1(C) in MIBC. All at *p*>0.05.


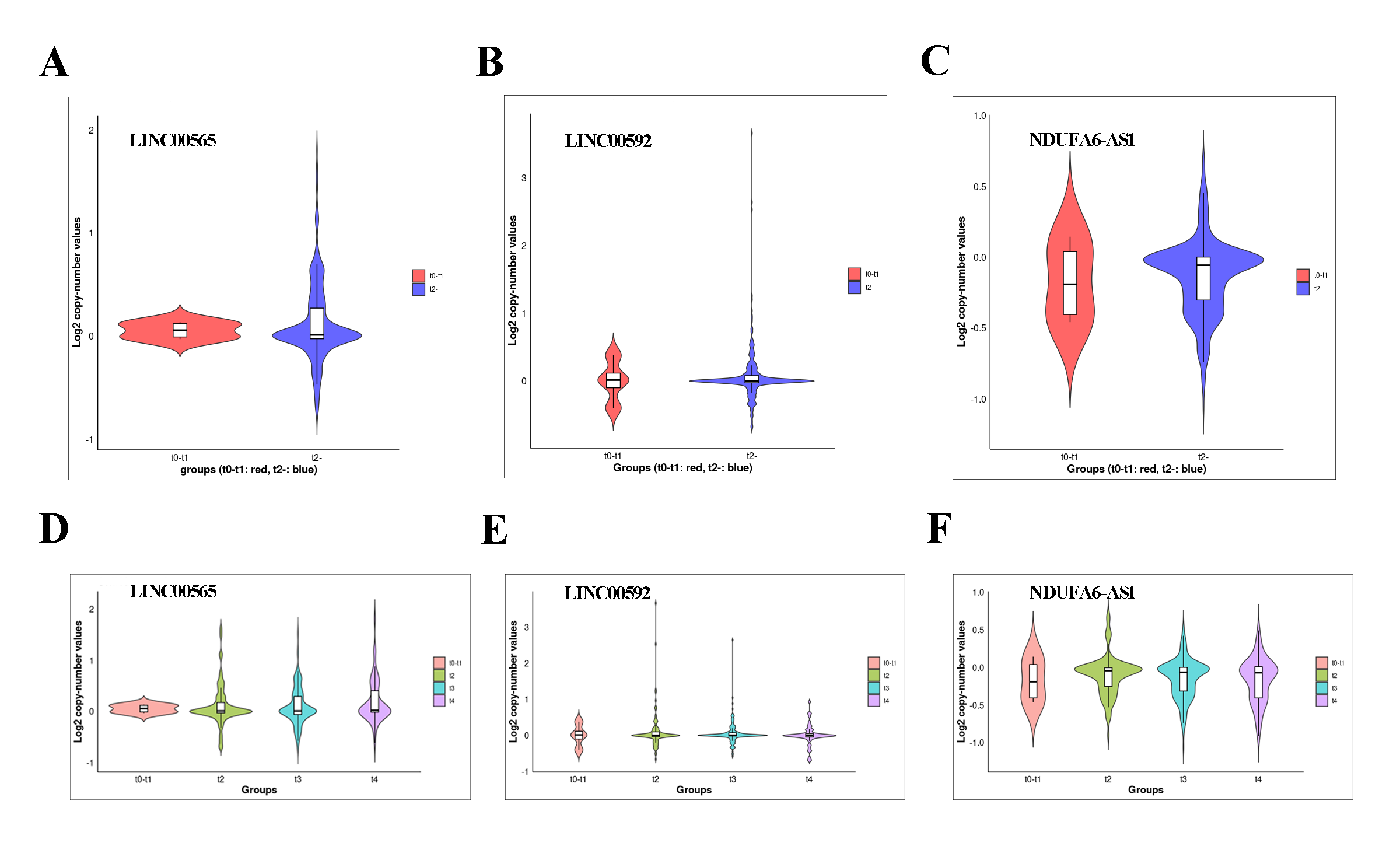


**Supplementary Figure S14**. Comparisons of copy number values of three lncRNAs in NMIBC and MIBC based on the data from Pan Cancer Atlas. (A-C) Analysis on copy number values of LINC00565(A), LINC00592(B), and NDUFA6-AS1(C) between NMIBC and MIBC. (D-F) Analysis on copy number values of LINC00565(D), LINC00592(E), and NDUFA6-AS1(F) among NMIBC and different stages of MIBC. All at *p*>0.05.

**SUPPLEMENTARY TABLES**

**Supplementary Table S1.** Primers of differentially expressed lncRNAs in MIBC used for RT-qPCR.

| **Gene** | **Primer** | **Sequencing** |
| --- | --- | --- |
| LINC00565 | Forward (5’-3’) | AGGAGATAGCCCAGGACTTGT |
|  | Reverse (5’-3’) | TCTCCACCCTCTTTCCATCCT |
| LINC00592 | Forward (5’-3’) | GCAGCAAGCCAGGAGAGATT |
|  | Reverse (5’-3’) | CTGCCTAACACGCACTTGGG |
| NDUFA6-AS1 | Forward (5’-3’) | ATGCCACCTCCTCCTTCTCT |
|  | Reverse (5’-3’) | CCCTGACCAGTGACGAGTTT |
| GAPDH | Forward (5’-3’) | GCACCGTCAAGGCTGAGAAC |
|  | Reverse (5’-3’) | TGGTGAAGACGCCAGTGGA |

**Supplementary Table S2.** Characteristics of study participants in the screening, training and validation sets.

| **Variable, N. (%)** | **Screening phase** | |  | **Training phase** | | |  |  |  | **Validation phase** | | |  |  |  | ***p**** |
| --- | --- | --- | --- | --- | --- | --- | --- | --- | --- | --- | --- | --- | --- | --- | --- | --- |
|  | **NMIBC** | **MIBC** | ***p*** | **Total** | **NMIBC** | **MIBC** |  | ***p*** |  | **Total** | **NMIBC** | **MIBC** |  | ***p*** |  |  |
| **Sex** | | | | | | | | | | | | | | | | |
| Male | 32 (80) | 34 (85) | 0.556 | 121 (67.2) | 55 (61.1) | 66 (73.3) |  | 0.081 |  | 81 (67.5) | 38 (63.3) | 43 (71.7) |  | 0.330 |  | 0.960 |
| Female | 8 (20) | 6 (15) |  | 59 (32.8) | 35 (38.9) | 24 (26.7) |  |  |  | 39 (32.5) | 22 (36.7) | 17 (28.3) |  |  |  |  |
| **Age** | | | | | | | | | | | | | | | | |
| <60 | 13 (32.5) | 8 (20) | 0.204 | 61 (33.9) | 33 (36.7) | 28 (31.1) |  | 0.431 |  | 30 (25) | 16 (26.7) | 14 (23.3) |  | 0.673 |  | 0.101 |
| ≥60 | 27 (67.5) | 32 (80) |  | 119 (66.1) | 57 (63.3) | 62 (68.9) |  |  |  | 90 (75) | 44 (73.3) | 46 (76.7) |  |  |  |  |
| **Tumor grade** | | | | | | | | | | | | | | | | |
| Low | 29 (72.5) | 25 (62.5) | 0.340 | 93 (51.7) | 52 (57.8) | 41 (45.6) |  | 0.101 |  | 60 (50.0) | 35 (58.3) | 25 (41.7) |  | 0.068 |  | 0.777 |
| High | 11 (27.5) | 15 (37.5) |  | 87 (48.3) | 38 (42.2) | 49 (54.4) |  |  |  | 60 (50.0) | 25 (41.7) | 35 (58.3) |  |  |  |  |
| **Tumor stage** | | | | | | | | | | | | | | | | |
| Ta | 12 (30.0) | - |  | 28 (15.6) | 28 (31.1) | - |  |  |  | 13 (10.8) | 13 (21.7) | - |  |  |  |  |
| T1 | 28 (70.0) | - |  | 62 (34.4) | 62 (68.9) | - |  |  |  | 47 (39.2) | 47 (78.3) | - |  |  |  |  |
| T2 | - | 13 (32.5) |  | 33 (18.3) | - | 33 (36.7) |  |  |  | 26 (21.7) | - | 26 (43.3) |  |  |  |  |
| T3 | - | 17 (42.5) |  | 38 (21.1) | - | 38 (42.2) |  |  |  | 20 (16.7) | - | 20 (33.3) |  |  |  |  |
| T4 | - | 10 (25.0) |  | 19 (10.6) | - | 19 (21.1) |  |  |  | 14 (11.7) | - | 14 (23.3) |  |  |  |  |
| **Lymph node metastasis** | | | | | | | | | | | | | | | | |
| Negative | 37 (92.5) | 36 (90) | 0.692 | 123 (68.3) | 64 (71.1) | 59 (65.6) |  | 0.423 |  | 91 (75.8) | 49 (81.7) | 42 (70.0) |  | 0.136 |  | 0.159 |
| Positive | 3 (7.5) | 4 (10) |  | 57 (31.7) | 26 (28.9) | 31 (34.4) |  |  |  | 29 (24.2) | 11 (18.3) | 18 (30.0) |  |  |  |  |
| **Vascular invasion** | | | | | | | | | | | | | | | | |
| Negative | 40 (100) | 39 (97.5) | 0.314 | 157 (87.2) | 81 (90.0) | 76 (84.4) |  | 0.264 |  | 106 (88.3) | 55 (91.7) | 51 (85) |  | 0.255 |  | 0.774 |
| Positive | 0 (0) | 1 (2.5) |  | 23 (12.8) | 9 (10.0) | 14 (15.6) |  |  |  | 14 (11.7) | 5 (8.3) | 9 (15) |  |  |  |  |
| **Tumor size** | | | | | | | | | | | | | | | | |
| <3cm | 38 (95) | 34 (85) | 0.136 | 147 (81.7) | 76 (84.4) | 71 (78.9) |  | 0.335 |  | 99 (82.5) | 52 (86.7) | 47 (78.3) |  | 0.230 |  | 0.854 |
| ≥3cm | 2 (5) | 6 (15) |  | 33 (18.3) | 14 (15.6) | 19 (21.1) |  |  |  | 21 (17.5) | 8 (13.3) | 13 (21.7) |  |  |  |  |

*p:* the differences between the NMIBC group and MIBC group in the screening set, the training set or the validation set.

*p**: the differences between the training set and the validation set.

**Supplementary Table S3.** Summary of draft reads of eight libraries by RNA-sequencing.

| **Sample** | **Raw reads** | **Clean reads** | **Mapped reads** | **Mapped ratio** |
| --- | --- | --- | --- | --- |
| **MIBC-1** | 84,939,948 | 84,912,500 | 76,509,427 | 90.07% |
| **MIBC-2** | 106,792,308 | 106,766,532 | 95,918,048 | 89.82% |
| **MIBC-3** | 87,107,534 | 87,080,882 | 78,309,255 | 89.90% |
| **MIBC-4** | 96,037,308 | 95,990,078 | 86,619,694 | 90.19% |
| **NMIBC-1** | 95,061,908 | 95,018,364 | 85,394,677 | 89.83% |
| **NMIBC-2** | 81,639,346 | 81,600,736 | 72,358,420 | 88.63% |
| **NMIBC-3** | 91,940,048 | 91,879,266 | 82,262,517 | 89.47% |
| **NMIBC-4** | 84,593,420 | 84,561,720 | 76,401,649 | 90.32% |

**Supplementary Table S4.** Summary of the top 10 most upregulated/downregulated intergenic lncRNA in chromosome 2/ chromosome 1 by RNA-sequencing.

| **Top 10**  **upregulated**  **Intergenic lncRNAs** | **Log_2_ FC** | **Gene**  **location** |  | **Top 10 downregulated intergenic lncRNAs** | **Log_2_ FC** | **Gene location** |
| --- | --- | --- | --- | --- | --- | --- |
| ENST00000425325 | 4.41 | chr 2 |  | ENST00000435574 | -4.08 | chr 1 |
| ENST00000564121 | 3.19 | chr 2 |  | TCONS_00001829 | -3.59 | chr 1 |
| TCONS_00003990 | 3.13 | chr 2 |  | ENST00000452366 | -2.93 | chr 1 |
| ENST00000436433 | 3.06 | chr 2 |  | uc001gqz.1 | -2.82 | chr 1 |
| TCONS_00004325 | 2.87 | chr 2 |  | TCONS_00001218 | -2.75 | chr 1 |
| TCONS_00003048 | 2.62 | chr2 |  | ENST00000412674 | -2.74 | chr 1 |
| ENST00000567151 | 2.49 | chr 2 |  | TCONS_00001254 | -2.63 | chr 1 |
| ENST00000430893 | 2.43 | chr 2 |  | TCONS_00001015 | -2.61 | chr 1 |
| ENST00000567718 | 2.27 | chr 2 |  | ENST00000564261 | -2.51 | chr 1 |
| ENST00000424321 | 2.18 | chr 2 |  | ENST00000427145 | -2.50 | chr 1 |

**Supplementary Table S5.** Diagnostic performance for MIBC of individual biomarkers and lncRNA-based panel in training and validation sets.

|  |  | **Cut-off value** | **Sensitivity** | **Specificity** | **AUC** |
| --- | --- | --- | --- | --- | --- |
|  | **Training set** |  |  |  |  |
|  | LINC00565 | 2.09 | 0.611 (0.503-0.712) | 0.956 (0.890-0.987) | 0.811 (0.746-0.865) |
|  | LINC00592 | 1.30 | 0.678 (0.571-0.772) | 0.744 (0.642-0.831) | 0.728 (0.657-0.792) |
|  | NDUFA6-AS1 | 1.85 | 0.589 (0.480-0.692) | 0.833 (0.740-0.904) | 0.766 (0.698-0.826) |
|  | lncRNA-based panel | -0.46 | 0.867(0.779-0.929) | 0.833(0.740-0.904) | 0.903 (0.850-0.942) |
|  | **Validation set** |  |  |  |  |
|  | LINC00565 | 1.80 | 0.617 (0.482-0.739) | 0.883 (0.774-0.952) | 0.787 (0.703-0.856) |
|  | LINC00592 | 1.51 | 0.533 (0.400-0.663) | 0.800 (0.677-0.892) | 0.664 (0.572-0.748) |
|  | NDUFA6-AS1 | 1.56 | 0.550 (0.416-0.679) | 0.900 (0.795-0.962) | 0.726 (0.637-0.803) |
|  | lncRNA-based panel | -0.20 | 0.817 (0.696-0.905) | 0.850 (0.734-0.929) | 0.875 (0.802-0.928) |

The optimal cut-off values for each factor were determined using Youden’s index.
